# EEG spectral and microstate analysis originating residual inhibition of tinnitus induced by tailor-made notched music training

**DOI:** 10.3389/fnins.2023.1254423

**Published:** 2023-12-11

**Authors:** Min Zhu, Qin Gong

**Affiliations:** ^1^Department of Biomedical Engineering, School of Medicine, Tsinghua University, Beijing, China; ^2^School of Medicine, Shanghai University, Shanghai, China

**Keywords:** tinnitus, tailor-made notched music training, residual inhibition, EEG, spectral analysis, microstate

## Abstract

Tailor-made notched music training (TMNMT) is a promising therapy for tinnitus. Residual inhibition (RI) is one of the few interventions that can temporarily inhibit tinnitus, which is a useful technique that can be applied to tinnitus research and explore tinnitus mechanisms. In this study, RI effect of TMNMT in tinnitus was investigated mainly using behavioral tests, EEG spectral and microstate analysis. To our knowledge, this study is the first to investigate RI effect of TMNMT. A total of 44 participants with tinnitus were divided into TMNMT group (22 participants; ECnm, NMnm, RInm represent that EEG recordings with eyes closed stimuli-pre, stimuli-ing, stimuli-post by TMNMT music, respectively) and Placebo control group (22 participants; ECpb, PBpb, RIpb represent that EEG recordings with eyes closed stimuli-pre, stimuli-ing, stimuli-post by Placebo music, respectively) in a single-blind manner. Behavioral tests, EEG spectral analysis (covering delta, theta, alpha, beta, gamma frequency bands) and microstate analysis (involving four microstate classes, A to D) were employed to evaluate RI effect of TMNMT. The results of the study showed that TMNMT had a stronger inhibition ability and longer inhibition time according to the behavioral tests compared to Placebo. Spectral analysis showed that RI effect of TMNMT increased significantly the power spectral density (PSD) of delta, theta bands and decreased significantly the PSD of alpha2 band, and microstate analysis showed that RI effect of TMNMT had shorter duration (microstate B, microstate C), higher Occurrence (microstate A, microstate C, microstate D), Coverage (microstate A) and transition probabilities (microstate A to microstate B, microstate A to microstate D and microstate D to microstate A). Meanwhile, RI effect of Placebo decreased significantly the PSD of alpha2 band, and microstate analysis showed that RI effect of Placebo had shorter duration (microstate C, microstate D), higher occurrence (microstate B, microstate C), lower coverage (microstate C, microstate D), higher transition probabilities (microstate A to microstate B, microstate B to microstate A). It was also found that the intensity of tinnitus symptoms was significant positively correlated with the duration of microstate B in five subgroups (ECnm, NMnm, RInm, ECpb, PBpb). Our study provided valuable experimental evidence and practical applications for the effectiveness of TMNMT as a novel music therapy for tinnitus. The observed stronger residual inhibition (RI) ability of TMNMT supported its potential applications in tinnitus treatment. Furthermore, the temporal dynamics of EEG microstates serve as novel functional and trait markers of synchronous brain activity that contribute to a deep understanding of the neural mechanism underlying TMNMT treatment for tinnitus.

## 1 Introduction

Tinnitus is a distressing condition characterized by a persistent perceived ringing or buzzing sound in the absence of any external sound source (Baguley et al., 2013). The prevalence of tinnitus has been reported to range from 6 to 22% (Axelsson and Ringdahl, 1989; Kim et al., 2015; Wu et al., 2015), and tends to increase with age (Oiticica and Bittar, 2015; Wu et al., 2015; McCormack et al., 2016), posing a significant social burden in an aging society. Unfortunately, there is currently no known cure for tinnitus, which is attributed to the great heterogeneity in the symptom characteristics of individuals (Cederroth et al., 2019).

Tinnitus is generally considered to be not only a consequence of cochlear damage, but also a symptom involving the plasticity of the central nervous system (Lanting et al., 2009; van Der Loo et al., 2009; Chen et al., 2016). The underlying mechanisms of tinnitus can be divided into three broad categories: (a) peripheral auditory afferentation and central maladaptive plasticity changes, (b) hyperactivity of spontaneous auditory neurons, and (c) increased cross-fiber synchronization between neurons (Eggermont and Roberts, 2012; Baguley et al., 2013). Neuroimaging, electrophysiology and invasive and non-invasive neuromodulation techniques are widely used in the study of chronic tinnitus (Claes et al., 2014; De Ridder and Vanneste, 2014; De Ridder et al., 2015a; Mohan et al., 2016, 2017; Hullfish et al., 2018a,b; Hullfish et al., 2019).

Pathological oscillatory activity in the brain has found considerable support in the theory of tinnitus etiology. In fact, differences in oscillatory activity within different frequency bands may indicate functional lesions, since focal low-frequency activity is often associated with abnormal brain function (Papanicolaou, 2009). Therefore, extensive research has been devoted to identifying neural correlates of conscious tinnitus perception by comparing spontaneous resting state brain activity oscillations in individuals with and without tinnitus, using non-invasive recording methods obtained in non-invasive silence, such as magnetoencephalography (MEG) or electroencephalography (EEG). In response to the theory of abnormal oscillatory behavior in tinnitus and its supporting evidence, many proposed treatments aim to directly or indirectly alter oscillatory activity with the aim of alleviating symptoms, such as transcranial magnetic stimulation (Londero et al., 2018; Dong et al., 2020; Pink et al., 2021), transcranial direct current stimulation (Santos et al., 2018; Wang et al., 2018; Labree et al., 2021), neurofeedback (Güntensperger et al., 2017; Kleinjung et al., 2018; Czornik et al., 2022), alternating current stimulation, transcranial random noise stimulation, transcutaneous vagus nerve stimulation, and bimodal stimulation (Langguth, 2020; Yakunina and Nam, 2021; Herrera-Murillo et al., 2022).

In 1971, Feldmann found that the presence of noise caused tinnitus symptoms to be suppressed after noise cancelation for about 1 min (Feldmann, 1971; Roberts et al., 2006; Roberts, 2007), a phenomenon known as “residual inhibition” (RI; Vernon, 1977; Henry and Meikle, 2000). RI can be observed with various types of sounds, including pure tones (Feldmann, 1971; Roberts et al., 2008; Reavis et al., 2012; Tyler et al., 2014; Neff et al., 2017, 2019), narrow-band noise (Roberts et al., 2008), amplitude-modulated sound (Reavis et al., 2012; Tyler et al., 2014; Neff et al., 2017, 2019; Schoisswohl et al., 2020), frequency-modulated sound (Reavis et al., 2012; Neff et al., 2017), white noise (WN) (Henry et al., 2004; Sedley et al., 2012, 2015), or broadband noise (Feldmann, 1971, 1983; Tyler et al., 1984; Kaltenbach, 2006; Roberts et al., 2006, 2008; Kahlbrock and Weisz, 2008; Fournier et al., 2018). RI is one of the few interventions that can temporarily inhibit tinnitus. Its potential as a valuable measure is evident in the clinic, especially as a diagnostic marker of subtypes and a prognostic indicator of an individual’s response to therapeutic acoustic stimuli. Consequently, RI proves to be an invaluable asset in investigating the intricate mechanisms underlying tinnitus and is expected to be a suitable technique to detect the resting-state neural networks underlying tinnitus perception (Sedley et al., 2015; Hu et al., 2019).

Sound therapy is a non-invasive method recommended for the treatment of tinnitus, as stated in the 2014 American Academy of Otolaryngology–Head and Neck Surgery Foundation (AO-HNSF) clinical practice guidelines (CPGs) (Tunkel et al., 2014). A novel and effective technique for non-invasive neuromodulation of the auditory cortex in patients with chronic tinnitus is tailor-made notched music training (TMNMT) (Pantev et al., 2012). In 2010, Okamoto et al. (2010) proposed TMNMT to modify pleasant music by filtering out an octave range of individual’s tinnitus frequency as the central frequency band, which strengthens lateral inhibition and inhibits the hyperactivity of auditory cortical neurons, thereby potentially eliminating or weakening tinnitus (Okamoto et al., 2010; Teismann et al., 2011, 2014; Pantev et al., 2014; Pape et al., 2014; Stein et al., 2015a,b, 2016).

Microstates refer to electric potential topographies recorded in a multichannel array across the scalp, that remain quasi-stable for 60–120 ms before rapidly transitioning to a different microstate (Michel and Koenig, 2018). In contrast to some other techniques, microstate analysis simultaneously considers signals from all electrodes to build a global representation of a functional state. The rich syntax of the microstate time series provides a range of novel quantifications of the EEG signal with potential neurophysiological relevance. Indeed, many studies have demonstrated that characteristics of the EEG microstate time series vary across behavioral states (Stevens and Kircher, 1998; Lehmann et al., 2010), personality types (Schlegel et al., 2012), consciousness (Bréchet and Michel, 2022), sleep (Peng et al., 2021), pain (May et al., 2021) and neuropsychiatric disorders, such as Alzheimer’s disease (Dierks et al., 1997; Hanoglu et al., 2022), Parkinson’s disease (Pal et al., 2021; Hanoglu et al., 2022), Huntington’s disease (Faber et al., 2021), schizophrenia (Lehmann et al., 2005; Kim et al., 2021), panic (Kikuchi et al., 2011), dementia (Lin et al., 2021). Multiple evidence suggest that the microstate time series may provide insights into the neural activity of the brain in the resting state (Britz et al., 2010; Musso et al., 2010; Yuan et al., 2012). Microstate analysis of EEG presents a potent, cost-effective, high-time resolution and clinically applicable neurophysiological approach to study and evaluate the global functional states of the brain in both health and disease.

To our knowledge, however, there is a lack of research examining the residual inhibition (RI) of tinnitus induced by tailor-made notched music training (TMNMT). Therefore, the objective of this study is to utilize scalp EEG recordings technology to investigate the phenomenon of RI in tinnitus by TMNMT, we aim to provide valuable experimental evidence and practical implications for the potential applications of TMNMT in tinnitus treatment.

## 2 Materials and methods

### 2.1 Participants and subgrouping

The study consisted of 44 participants recruited from the faculty and students of Tsinghua University, who had been experiencing tinnitus for a duration of more than 6 months. During the recruitment process, we have established specific requirements for potential subjects beyond tinnitus, including proficiency in language expression, as well as the ability to identify and comprehend frequency and loudness. Certain exclusion criteria were applied, included Meniere’s disease, chronic ear infections, otosclerosis, tumors, mental disorders, a history of drug/alcohol abuse, chronic headaches, and pulsating tinnitus. Eligible participants, with 22 in each group of TMNMT (nm) and Placebo (pb), were kept unaware of their group assignment as part of a single-blind study, using a prespecified label. A participant in TMNMT group who was unable to identify and comprehend the frequency and loudness of tinnitus was reassigned to Placebo group. Prior to conducting the study, the Medical Ethics Committee of Tsinghua University granted approval, and all participants provided their informed consent.

### 2.2 Audiological and behavioral assessments

All participants underwent a pure-tone hearing test, which obtained hearing thresholds of 125, 250, 500, 1,000, 2,000, 3,000, 4,000, 6,000 and 8,000 Hz according to the procedures prescribed by the British Academy of Audiology. The mean hearing threshold of all participants was below 70 dB HL. The mean hearing threshold was calculated as the average of the hearing thresholds at 500, 1000, 2,000 and 4,000 Hz (Dispenza et al., 2011; Bing et al., 2018). Furthermore, Tinnitus Handicap Inventory (THI), Tinnitus functional index (TFI), and Visual Analogue Scale (VAS, included Loudness and Annoyance) were employed to evaluate the distress and impact of tinnitus-related on participants’ daily lives.

### 2.3 Tinnitus pitch and loudness matching tests

Tinnitus pitch and loudness matching tests were conducted using a portable instrument named QingEr Tinnitus Treatment Instrument (QEHS-TI01, designed and manufactured by Wuxi QingEr HuaSheng Technology Co., LTD). A comprehensive set of 188 pure tone frequencies ranging from 125 Hz to 8.5 kHz, divided by 1/30 octave intervals, was utilized to match the pitch of the participants’ tinnitus (Gong et al., 2022). The participants were asked to compare the tones they heard with the pitch of individual tinnitus until they achieved a tone that exactly matched their tinnitus pitch. Following confirming the tinnitus pitch, an approximate tinnitus loudness level was given using the same instrument, which the level was adjusted in 1 dB sound pressure level (SPL) steps until participants reported that the loudness accurately matched their perception of tinnitus loudness (Kim et al., 2016). This process allowed for the fine-tuning of the loudness level to align with the subjective experience of each participant.

### 2.4 Experimental pipeline

**Figure 1:**
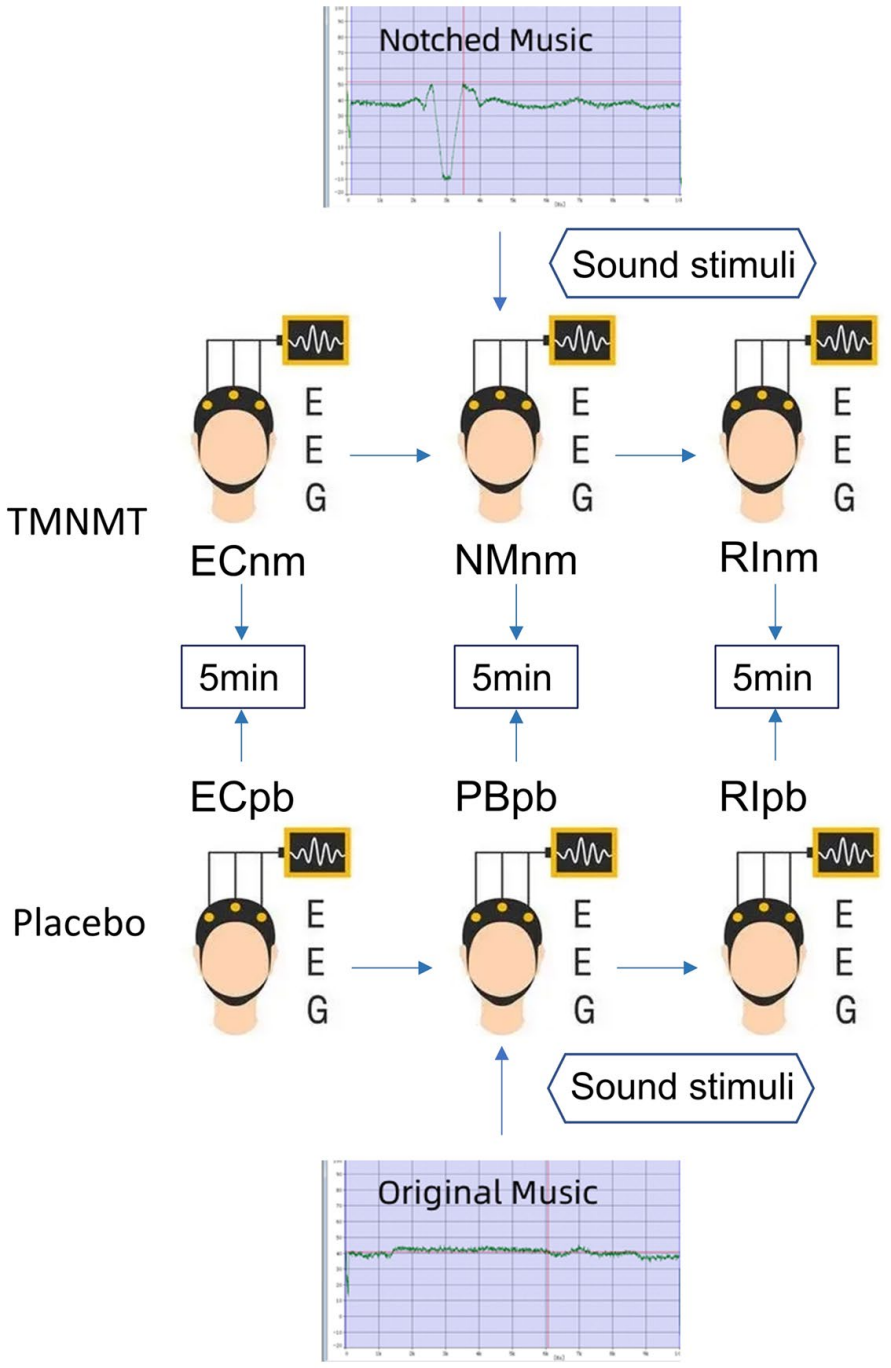
Experimental pipeline. ECnm, NMnm, RInm subgroups in TMNMT group and ECpb, PBpb, RIpb subgroups in Placebo group. ECnm, EEG recordings with eyes closed stimuli-pre by TMNMT music; NMnm, EEG recordings with eyes closed stimuli-ing by TMNMT music; RInm, EEG recordings with eyes closed stimuli-post by TMNMT music; ECpb, EEG recordings with eyes closed stimuli-pre by Placebo music; PBpb, EEG recordings with eyes closed stimuli-ing by Placebo music; RIpb, EEG recordings with eyes closed stimuli-post by Placebo music.

In TMNMT group, the participants underwent a series of EEG recordings during different stages of the experiment. Initially, a 5-min resting-state EEG with eyes closed (ECnm subgroup) was recorded. Following this, there was a brief period of rest with eyes open. Subsequently, a 5-min segment of notched music with a loudness of 10 dB SPL above the threshold of tinnitus loudness was played by QEHS-TI01, which specifically filtered out the tinnitus central frequency and increased the lateral inhibition effect on the edge frequency bands of the notch area, associated with each participant’s individual tinnitus. To better target tinnitus participants with notched music, the music energy spectrum was equalized by digitally redistributing energy from lower to higher-frequency ranges, a process often referred to as “flattening” (Teismann et al., 2011), thereby enriching the energy spectrum in the higher-frequency range (Figure 1). The music was delivered to the both ears via a pair of insert earphones. The EEG data was simultaneously recorded with eyes closed at the same time (NMnm subgroup). Finally, another 5-min EEG recording was conducted with eyes closed, known as residual inhibition (RInm subgroup).

In contrast, Placebo group, as control group, underwent a similar procedure but with slight variations. The participants in Placebo group also underwent a 5-min resting-state EEG with eyes closed (ECpb subgroup). However, instead of the notched music intervention, a 5-min segment of original music with a loudness of 10 dB SPL above the threshold of tinnitus loudness without any specific processing was played by QEHS-TI01. The EEG data was recorded simultaneously with eyes closed during this period (PBpb subgroup). Similar to TMNMT group, a subsequent 5-min EEG recording for the residual inhibition (RIpb subgroup) was conducted with eyes closed. A schematic representation of these procedures can be seen in Figure 1.

TMNMT and Placebo music were derived from the nature environmental sounds (Strauss et al., 2017), such as rain, streams, wind and waves, etc., which were sounds comfortable and relaxed. The distinction between TMNMT music and Placebo music is subtle, and participants were unable to discern their respective groups based solely on the auditory stimuli they received.

### 2.5 EEG data collection and pre-processing

The electroencephalography (EEG) data were acquired using the Neuroscan system^1^ in a quiet room, with each participant seated in a comfortable chair in an upright position. To minimize potential interference from alcohol, coffee, cola, and tea on EEG recordings (Barry et al., 2011; Foxe et al., 2012; Vanneste and De Ridder, 2012a), participants were instructed to abstain from consuming these substances for 24 h prior to the study. Cleaned scalp with a scrub to ensure optimal signal quality before recording baseline EEG. The 64-channel Neuroscan synnamps2 Quick Cap placed in the international standard 10–20 system was utilized to collect EEG, and the EEG signals was sampled at a rate of 1,000 Hz using a Neuroscan synnamps2 amplifier to check that the impedance levels remained below 5 kΩ throughout the recording session. The midline reference point was located at the vertex (Cz), and the ground electrode was located at the AFZ.

The acquired EEG data were preprocessed using MATLAB and EEGLAB. The preprocessing steps consisted of the following procedures: Removal of irrelevant electrodes and resampling the data to 512 Hz. a Finite Impulse Response (FIR) filter performed for bandpass filtering in the frequency range of 0.5 to 100 Hz, with additional notch filtering at 50 Hz and 100 Hz, re-selected average reference and divided the data into 2 s epochs, identified and rejected the bad epochs via visual inspection, including instances of swallowing, gnashing, chewing and excessive muscle artifacts. Subsequently, performed a spherical interpolation algorithm to interpolate the removed channels, to ensure that have an equal number of channels for all participants. Data from the remaining electrodes were performed using the infomax algorithms for independent component analysis (ICA) (Jung et al., 1998) to remove eye blinks, saccades, muscle artifacts, electrocardiogram (ECG) signals, head movements, channel noise, and other transient noises. Artifact detection was detected on all epochs by a voltage threshold of ±100 μV, and any epochs not within this threshold were excluded.

These preprocessing steps helped ensure the data’s quality and remove unwanted artifacts, allowing for more accurate subsequent analysis and interpretation.

### 2.6 Spectral analysis

Spectral analysis was performed by Welch’s method. The Welch’s method involves dividing the signal of N samples into K data segments of M samples, with an overlap of D samples. If D = M/2, the overlap is 50%; while D = 0, the overlap is 0%. The overlapping segments are then windowed (i.e., multiplied by a symmetric bell-shaped window). Subsequently, the Discrete Fourier Transform (DFT) is computed for each windowed data segment, resulting in the periodogram. The final spectral estimate is obtained by averaging the periodograms of all data segments. The Welch’s method can lead to a reduction in the variance of spectral estimate as 1/K. The power spectral density [PSD, 10 log10 (V^2^/Hz, dB)] for each channel based on the periodogram was calculated and averaged across channels to measure comparisons between groups in each frequency band.

Spectral estimation was applied on the EEG recordings in a specific time period to calculate the power of various certain rhythms: delta (0.5 ~ 4 Hz), theta (4 ~ 8 Hz), alpha1 (8 ~ 10 Hz), alpha2 (10 ~ 13 Hz), beta1 (13 ~ 18 Hz), beta2 (18 ~ 21 Hz), beta3 (21 ~ 30 Hz), gamma1 (30 ~ 45 Hz), gamma2 (55 ~ 100 Hz). By examining the power values within these frequency bands, comparisons between different groups could be made, shedding light on potential differences in neural activity.

### 2.7 Microstate analysis

Microstate analysis can be considered as a method to reveal the topographic steady period (80–120 ms) from EEG signals. In fact, applying spatial reduction using a clustering algorithm allows extraction of a certain number of fixed topologies (global templates), called microstates. By fitting such topographic maps to the EEG time processes, it is possible to obtain a discrete sequence of microstates (Prete et al., 2022). Microstate analysis aims at identifying major topographic configurations (global templates or microstates) that alternate over the course of EEG time to describe the ongoing brain dynamics (Khanna et al., 2015). By quantitative measures, characteristics characterizing the specific sequences of microstates can be calculated.

**Figure 2:**
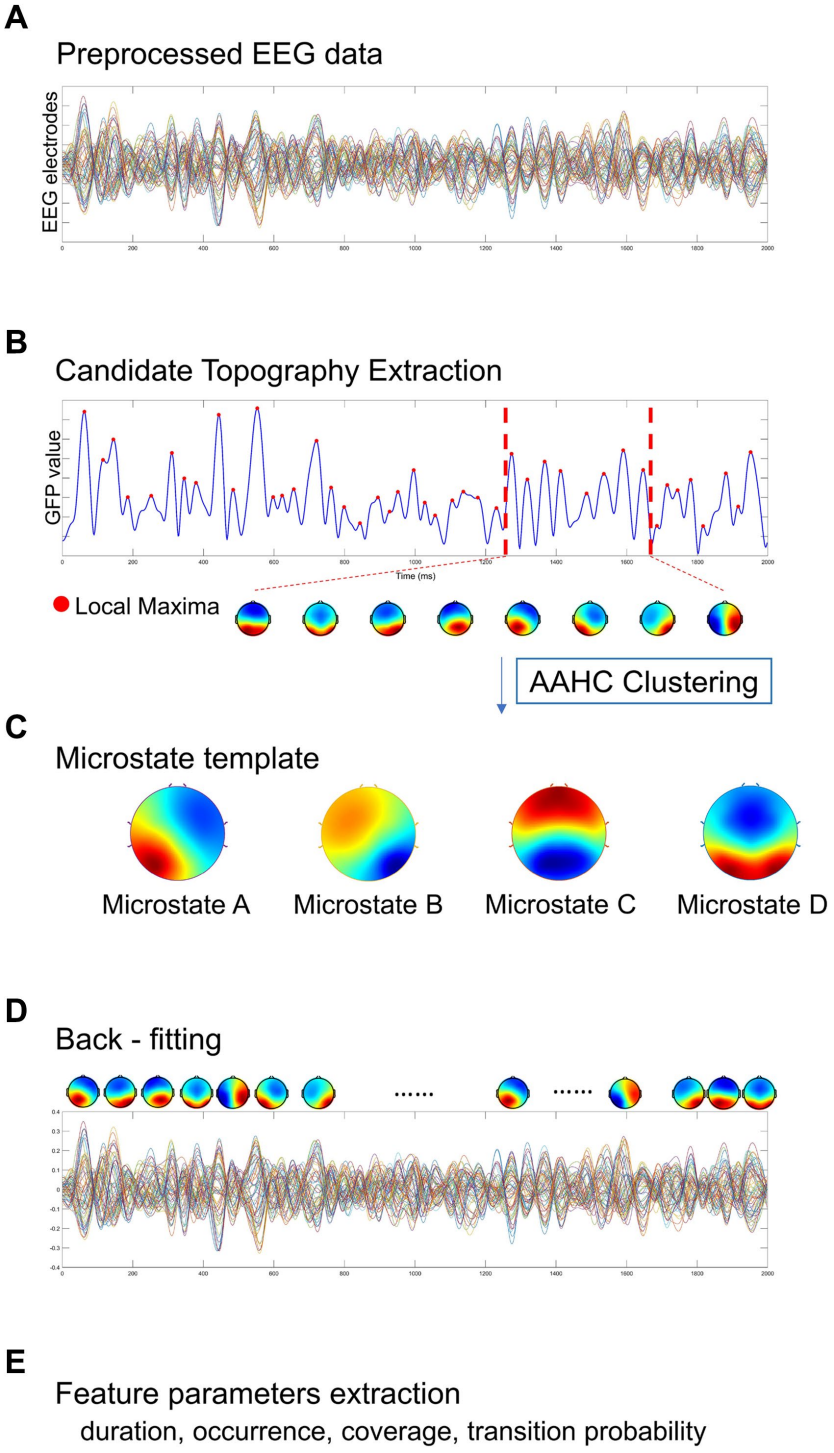
A standard procedure of EEG microstate analysis. Based on **(A)** the preprocessed EEG data, **(B)** candidate topographies with high signal-to-noise ratios were extracted from the local maxima of the GFP curve, **(C)** four templates were obtained after AAHC Clustering. **(D)** The final detected EEG microstate templates were then fitted back into the preprocessed EEG data by assigning each time point to a predominant microstate. After EEG microstates back-fitting, the original EEG time series were re-represented into EEG microstate sequences covering whole-brain spontaneous spatial–temporal activities. **(E)** A several of microstate feature metrics were calculated for quantitative measurement, including duration, occurrence, coverage, transition probability.

The Microstate analysis was conducted using EEGLAB plug-in^2^ for artifact-free EEG data analysis. The EEG data performed digital bandpass filtering in the range of 2 ~ 20 Hz (Koenig et al., 2002; Kindler et al., 2011; Nishida et al., 2013). A resting-state EEG microstate analysis is implemented as shown in Figure 2, including global field power extraction, microstate clustering, back-fitting (Pascual-Marqui et al., 1995). EEG microstate analysis begins with a bottom-up extraction of EEG microstate templets from the spontaneous EEG signals (Figures 2A,C). Then, a top-down process termed back-fitting (Figure 2D) is conducted to re-represent the EEG data into a series of dynamic microstate sequences. The global field power (GFP) of multi-channel EEG data represents global brain activity, which is a real-time, reference-independent measure of whole brain neuronal activity (Lehmann and Skrandies, 1980). The EEG topography is stable around the peak of GFP (Koenig et al., 2002). Therefore, EEG data were first simplified to the time point at the local maximum of global field power (GFP), representing the time point at the highest signal-to-noise ratio (Murray et al., 2008; Khanna et al., 2015; Michel and Koenig, 2018). The GFP reflects the potential variances across multiple electrodes at a specific point in time. The timepoints between two GFP peaks were obtained using nearest-neighbor interpolation. GFP can be calculated by the following formula:


$$GFP\left(t\right)=\sqrt{\frac{{\left[\sum_{i}^{N}\left(Vi\;\left(t\right)\;-\mathrm{Vmean}\left(t\;\right)\right)\right]}^{2}}{N}}$$

Where *N* represents the number of electrodes in the EEG data (*i* = 1: 60), *V* represents the electrical potential measured over the scalp. *Vi*(*t*) represents the instantaneous electrical potential at electrode *i* and time *t*, and *V*mean(*t*) represents the average electrical potential at all electrodes at time *t*. The global field power time series (GFP) is calculated as the spatial standard deviation of the EEG topography at each given time to quantify synchronous activity across all the electrodes at each timepoint (Figure 2B) (Skrandies, 1990). At the local GFP maximum (red dots), the spatial configuration of the EEG is considered stable and accounts for most of the variance of the time series (Wackermann et al., 1993), and switch to the next topographic map immediately after the GFP reaches a minimum peak (Lehmann et al., 1987). In microstate analysis, the topographies of GFP peaks are considered to be discrete microstates, whereas dynamic changes in EEG signals as variations of these states (Khanna et al., 2015). The atomize-agglomerate hierarchical cluster (AAHC), a modified k-means that provides a unique cluster for microstate analysis, was used to generate EEG topological clusters (Tibshirani and Walther, 2005; Murray et al., 2008; Britz et al., 2009). In the process of clustering, the algorithm ignores EEG polarity (Schlegel et al., 2012; Kuhn et al., 2015; Pipinis et al., 2017). Cluster analysis is conducted first at the individual template maps level and then at group level, and determine the optimal number of clustering based on cross validation (CV) criterion and Krzanowski-Lai (KL) criteria (Murray et al., 2008). Previous researches have suggested that EEG can typically be clustered into four distinct microstates, categorized as classes A, B, C, and D (Brodbeck et al., 2012; Khanna et al., 2015).

In this study, we categorized the microstates of each subject and each group. Firstly, the EEG maps of all participants were clustered into four grand subsets from all groups (TMNMT and Placebo). With the mean map of each subset computed, the four grand subsets were assigned to classes A, B, C, and D, respectively. Then, the EEG maps within each group were clustered into four group subsets. Each group subsets were assigned to the same class as the grand subset that exhibited the highest similarity. Finally, the EEG maps of each subject from TMNMT and Placebo groups were clustered and assigned to one of the four microstate classes. Spatial correlations were calculated between each map at group level and the topographies (maps) at the GFP peaks of the original EEG signals at individual level. Therefore, microstate maps were used to determine the back-fitting to the original map topography at each GFP peak according to maximum spatial correlation.

With the obtained microstates sequence, we conducted the computation and analysis of several metrics that hold potential neurophysiological significance (Figure 2E):

1. mean duration (ms) of a microstate class: defined as the average time covered by the same microstate class (Michel and Koenig, 2018).
2. occurrence rate (/s) per second of a microstate class: defined as the number of occurrences of a given microstates class per second across all analyzed epochs (Michel and Koenig, 2018).
3. time coverage (%) of a microstate class: defined as the percentage of the total analysis time in a given microstate class (Lehmann et al., 1987, 2005; Andreou et al., 2014; Khanna et al., 2015; Seitzman et al., 2017).
4. transition probability (%) between microstate classes: defined as the probability of transition from one given class to another one (Khanna et al., 2015; Michel and Koenig, 2018). The transition probability of a microstate means that the microstate is non-random and has the potential significance of sequence transition (Lehmann et al., 2005).

### 2.8 Statistical analysis

Continuous variables were compared between the two groups using an independent sample t-test, and gender distribution was compared using Pearson’s *χ*^2^ test. To compare the power spectral density (PSD), repeated measures analysis of variance (RM-ANOVA) and two-tailed paired t test were performed separately for each group, with corrected effects for multiple comparisons of the false discovery rate (FDR). RM-ANOVA was also applied to analyze inter-group differences, with microstate classes (microstate A, B, C, and D) and microstate metrics (duration, occurrence, coverage, transition probabilities) as within-subject factors and group as between-subject factors. In case where significant group main effects or interactions were found in the RM-ANOVA, univariate ANOVA was then performed to investigate the simple effects. FDR correction was performed to adjust for multiple comparisons.

## 3 Results

### 3.1 Demographics and clinical characteristics

There were no significant differences in terms of gender, age, mean hearing threshold, tinnitus duration, tinnitus lateralization, THI score, TFI score, and VAS score between TMNMT group and Placebo group. Demographics and clinical characteristics were given in Table 1.

**Table 1:** Demographics and clinical characteristic of two groups.

Variable	TMNMT	Placebo	*p*-value
Gender (n, male/female)	22 (11/11)	22 (12/10)	>0.999
Age (year, std)	39.18 ± 20.99	37.73 ± 16.67	0.805
Mean hearing threshold (HL) Left/Right ear (std)	22.33 ± 17.54/21.02 ± 17.40	18.01 ± 11.44/16.5 ± 10.73	0.351/0.321
Tinnitus duration (year, std)	3.92 ± 3.15	3.87 ± 2.24	0.949
Tinnitus lateralization (left/right/bilateral)	7/6/9	7/5/10	>0.999
THI score (std)	38.50 ± 19.15	38.7 ± 15.96	0.975
TFI score (std)	94.76 ± 61.60	94.45 ± 52.08	0.986
VAS score (std)	4.85 ± 2.10	4.77 ± 1.91	0.894

A THI score of 38 and a TFI score of 50 are generally considered as cutoff values indicative of tinnitus-related high distress.

### 3.2 Behavioral tests

**Figure 3:**
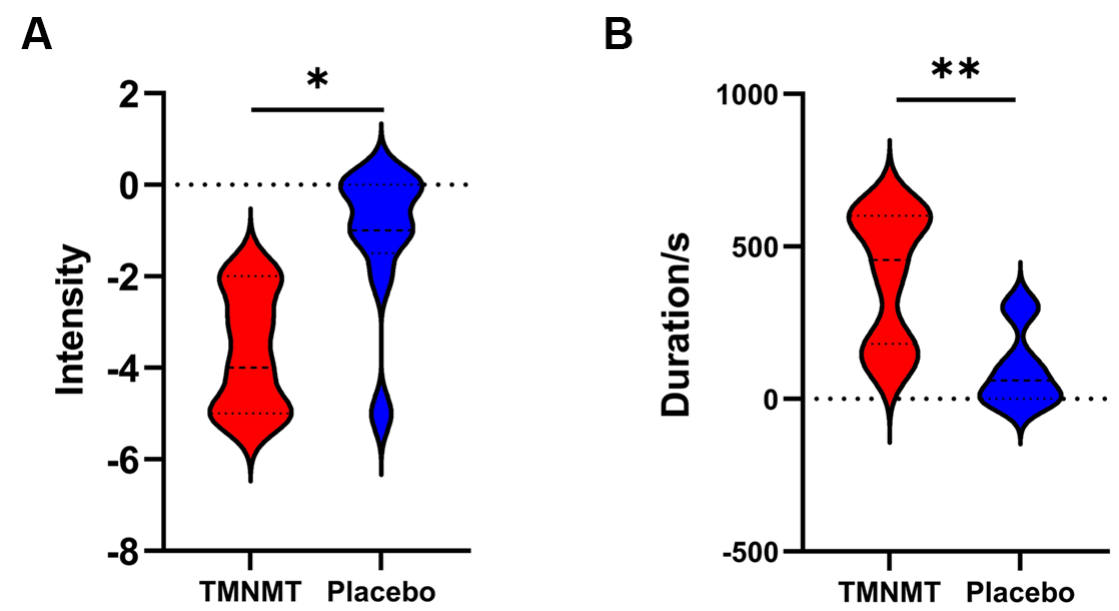
The intensity and duration of residual inhibition (RI) between TMNMT and Placebo groups. **(A)** TMNMT: −3.6 ± 1.3; Placebo: −1.1 ± 1.6, *p* = 0.0257; **(B)** TMNMT: 403 ± 215 s; Placebo: 96 ± 123 s, *p* = 0.0045; *p < 0.05, **p < 0.01.

A Likert scale was used to assess the effectiveness of TMNMT and Placebo interventions in tinnitus inhibition. The scale ranged from −5 to 5 (−5 indicates that tinnitus disappears after the sound stimuli; 0 indicates that no change in tinnitus loudness; and 5 indicates that tinnitus loudness doubled). We also recorded the duration of residual inhibition (RI), which spanned from the end of the sound stimuli until the tinnitus loudness returned to its initial level. The results showed TMNMT had a significantly stronger inhibition intensity (TMNMT: −3.6 ± 1.3; Placebo: −1.1 ± 1.6, *p* = 0.0257; Figure 3A) and longer inhibition time (TMNMT: 403 ± 215 s; Placebo: 96 ± 123 s, *p* = 0.0045; Figure 3B) compared to Placebo group by independent samples t test.

### 3.3 Whole-brain spectral analysis

**Figure 4:**
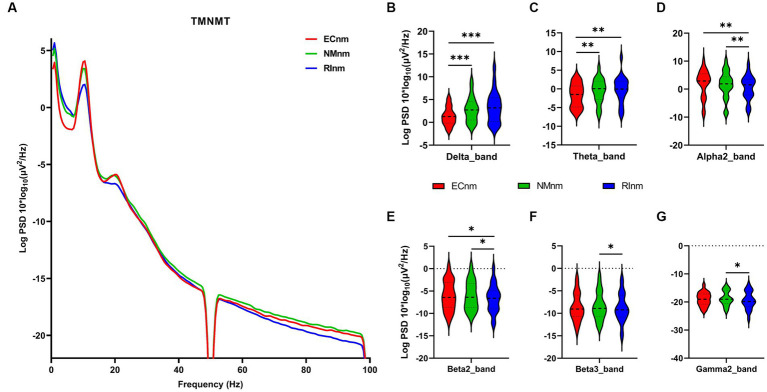
Waveforms of spectral analysis in TMNMT group. **(A)** The power spectral density (PSD) waveform of full frequency bands in ECnm, NMnm, RInm subgroups of TMNMT group. **(B–G)** The PSD of delta **(B)**, theta **(C)**, alpha2 **(D)**, beta2 **(E)**, beta3 **(F)**, gamma2 **(G)** bands in ECnm, NMnm, RInm subgroups of TMNMT group. ECnm, EEG recordings with eyes closed stimuli-pre by TMNMT music; NMnm, EEG recordings with eyes closed stimuli-ing by TMNMT music; RInm, EEG recordings with eyes closed stimuli-post by TMNMT music. *p < 0.05, **p < 0.01, ***p < 0.001.

**Figure 5:**
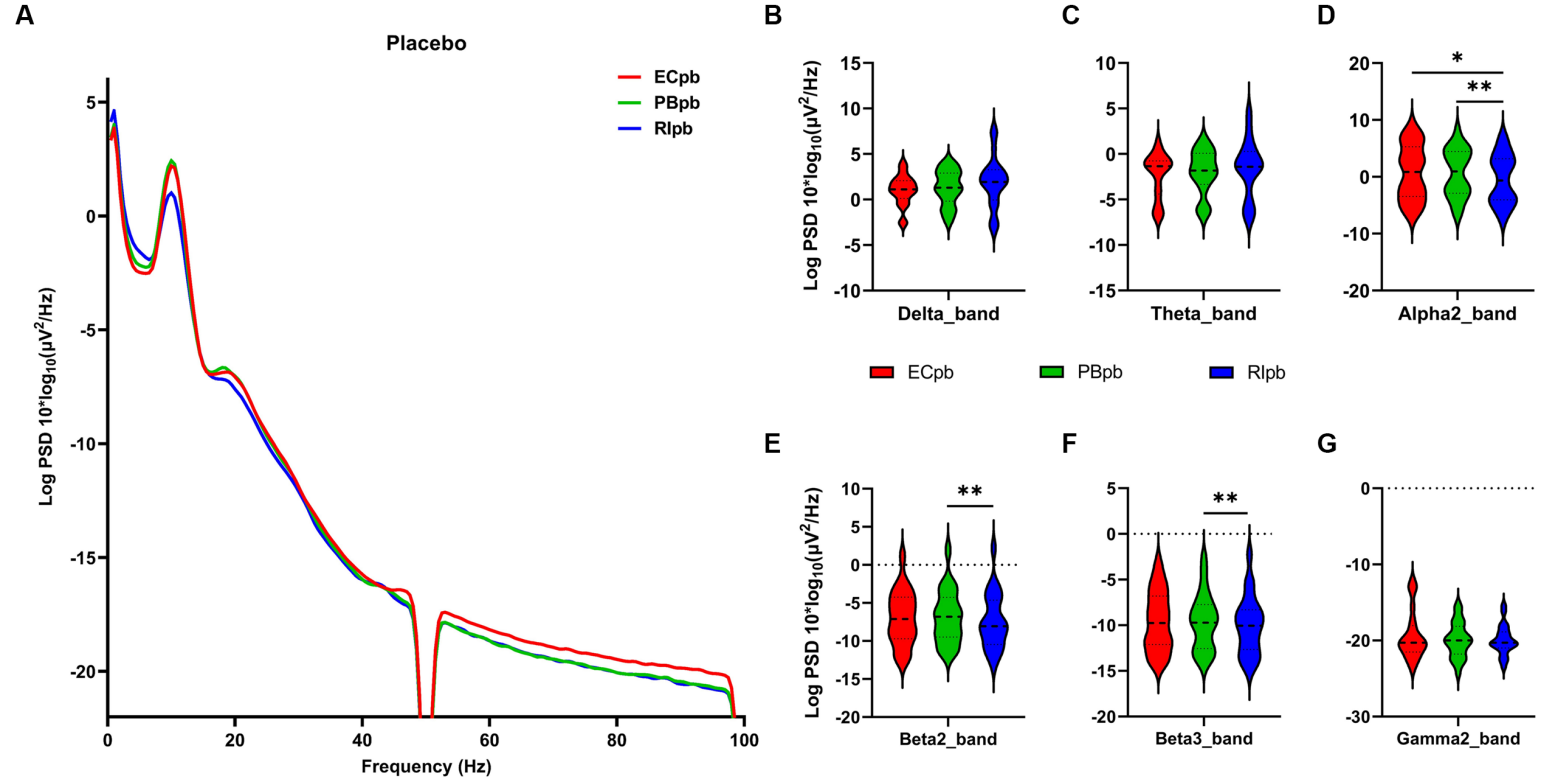
Waveforms of spectral analysis in Placebo group. **(A)** The power spectral density (PSD) waveform of full frequency bands in ECpb, PBpb, RIpb subgroups of Placebo group. **(B–G)** The PSD of delta **(B)**, theta **(C)**, alpha2 **(D)**, beta2 **(E)**, beta3 **(F)**, gamma2 **(G)** bands in ECpb, PBpb, RIpb subgroups of Placebo group. ECpb, EEG recordings with eyes closed stimuli-pre by Placebo music; PBpb, EEG recordings with eyes closed stimuli-ing by Placebo music; RIpb, EEG recordings with eyes closed stimuli-post by Placebo music. *p < 0.05, **p < 0.01.

The comparison of spectral analysis of the two groups of participants stimuli-pre, stimuli-ing and stimuli-post of different modulated music by repeated measures ANOVA showed different significant differences. The frequency band (delta, theta, alpha1, alpha2, beta1, beta2, beta3, gamma1, gamma2) was regarded as a within-subject factor and group (ECnm, NMnm, RInm, ECpb, PBpb, RIpb) as a between-subject factor. From an analysis of the waveforms, Figure 4A showed the power spectral density (PSD) of whole-brain full bands in TMNMT group, the results of PSD showed that the main effect of frequency band factor (*F* = 300.7; *p* < 0.0001) was significant, while the Frequency band ∗ Group interaction effect (*F* = 0.5583; *p* = 0.9146) and the main effect of group factor were not significant (*F* = 0.7915; *p* = 0.4537). Post-hoc comparisons showed the PSD of delta band (ECnm vs. NMnm, *p* = 0.0005; ECnm vs. RInm, *p* = 0.0005; Figure 4B) and theta band (ECnm vs. NMnm, *p* = 0.0011; ECnm vs. RInm, *p* = 0.0043; Figure 4C) were significantly increased; alpha2 band (ECnm vs. RInm, *p* = 0.0026; NMnm vs. RInm, *p* = 0.0027; Figure 4D), beta2 band (ECnm vs. RInm, *p* = 0.0431; NMnm vs. RInm, *p* = 0.0251; Figure 4E), beta3 band (NMnm vs. RInm, *p* = 0.0234; Figure 4F) and gamma2 band (NMnm vs. RInm, *p* = 0.0180; Figure 4G) were significantly decreased (Table 2 and Figure 4). Figure 5A showed the PSD of whole-brain full bands in Placebo group, the results of PSD showed that the main effect of frequency band factor (*F* = 315.2; *p* < 0.0001) was significant, while the Frequency band ∗ Group interaction effect (*F* = 0.2623; *p* = 0.9984) and the main effect of group factor were not significant (*F* = 0.3160; *p* = 0.7292). Post-hoc comparisons showed the PSD of alpha2 band (ECpb vs. RIpb, *p* = 0.0352; PBpb vs. RIpb, *p* = 0.0033; Figure 5D), beta2 band (PBpb vs. RIpb, *p* = 0.0088; Figure 5E) and beta3 band (PBpb vs. RIpb, *p* = 0.0027; Figure 5F) were significantly decreased in Placebo group (Table 3 and Figure 5).

**Table 2:** The power spectral density (PSD) of whole-brain full bands in TMNMT group (ECnm, NMnm, RInm).

PSD (μV^2^/Hz) Mean (Std)	ECnm (*n* = 22)	NMnm (*n* = 22)	RInm (*n* = 22)	*t*-value/*p*-value (ECnm – NMnm)	*t*-value/*p*-value (ECnm – RInm)	*t*-value/*p*-value (NMnm – RInm)
Delta	1.30 (1.94)	2.80 (2.66)	3.29 (3.37)	3.9950/0.0005	3.8830/0.0005	1.2870/0.0743
Theta	−1.75 (3.08)	−0.50 (3.20)	−0.21 (3.50)	3.7950/0.0011	2.9200/0.0043	1.0020/0.1146
Alpha1	1.60 (5.24)	1.60 (4.86)	0.81 (4.37)	0.0012/0.9990	1.7810/0.0894	1.9900/0.0598
Alpha2	1.97 (4.74)	1.54 (4.66)	0.55 (4.21)	0.9125/0.1302	3.4390/0.0026	3.1220/0.0027
Beta1	−5.58 (3.06)	−5.33 (3.00)	−5.57 (2.70)	1.7250/0.3126	0.0689/0.9930	0.9059/0.5910
Beta2	−6.03 (3.19)	−6.04 (3.11)	−6.69 (3.01)	0.0295/0.6837	2.1770/0.0431	2.7510/0.0251
Beta3	−8.66 (3.01)	−8.40 (3.02)	−8.82 (3.12)	1.4290/0.2639	0.6472/0.5508	2.4450/0.0234
Gamma1	−13.75 (2.42)	−13.34 (2.45)	−13.64 (2.84)	1.9570/0.2009	0.4779/0.6696	1.5010/0.2333
Gamma2	−19.00 (2.35)	−18.72 (2.52)	−19.45 (3.05)	0.9670/0.3446	1.0850/0.2903	2.9000/0.0180

ECnm, EEG recordings with eyes closed stimuli-pre by TMNMT music; NMnm, EEG recordings with eyes closed stimuli-ing by TMNMT music; RInm, EEG recordings with eyes closed stimuli-post by TMNMT music.

**Table 3:** The power spectral density (PSD) of whole-brain full bands in Placebo group (ECpb, PBpb, RIpb).

PSD (μV^2^/Hz) Mean (Std)	ECpb(*n* = 22)	PBpb(*n* = 22)	RIpb(*n* = 22)	*t*-value/*p*-value (ECpb – PBpb)	*t*-value/*p*-value (ECpb – RIpb)	*t*-value/*p*-value (PBpb – RIpb)
Delta	1.04 (1.63)	1.31 (1.90)	1.89 (2.72)	1.2150/0.2380	1.7930/0.0874	1.3420/0.1939
Theta	−2.34 (2.43)	−2.02 (2.41)	−1.62 (3.11)	1.9760/0.1920	1.6120/0.1219	1.0390/0.3262
Alpha1	0.53 (4.84)	0.94 (4.51)	0.03 (4.78)	1.1440/0.2654	0.9287/0.3636	2.4150/0.0785
Alpha2	0.74 (4.75)	0.67 (4.15)	−0.46 (4.30)	0.1880/0.5969	2.2750/0.0352	3.6290/0.0033
Beta1	−5.95 (3.10)	−5.99 (2.88)	−6.12 (3.10)	0.1660/0.8697	0.5470/0.5902	0.6422/0.5277
Beta2	−6.97 (3.46)	−6.87 (3.42)	−7.41 (3.58)	0.4176/0.4764	1.8020/0.0903	3.2130/0.0088
Beta3	−9.56 (3.22)	−9.63 (3.04)	−10.00 (3.15)	0.3574/0.2535	2.3540/0.0852	3.8650/0.0027
Gamma1	−14.68 (2.72)	−14.81 (2.60)	−14.91 (2.61)	0.5475/0.5898	0.9301/0.3629	0.6399/0.5291
Gamma2	−19.38 (3.13)	−19.93 (2.24)	−19.95 (1.91)	1.3990/0.1764	1.4270/0.1684	0.0707/0.9443

ECpb, EEG recordings with eyes closed stimuli-pre by Placebo music; PBpb, EEG recordings with eyes closed stimuli-ing by Placebo music; RIpb, EEG recordings with eyes closed stimuli-post by Placebo music.

**Figure 6:**
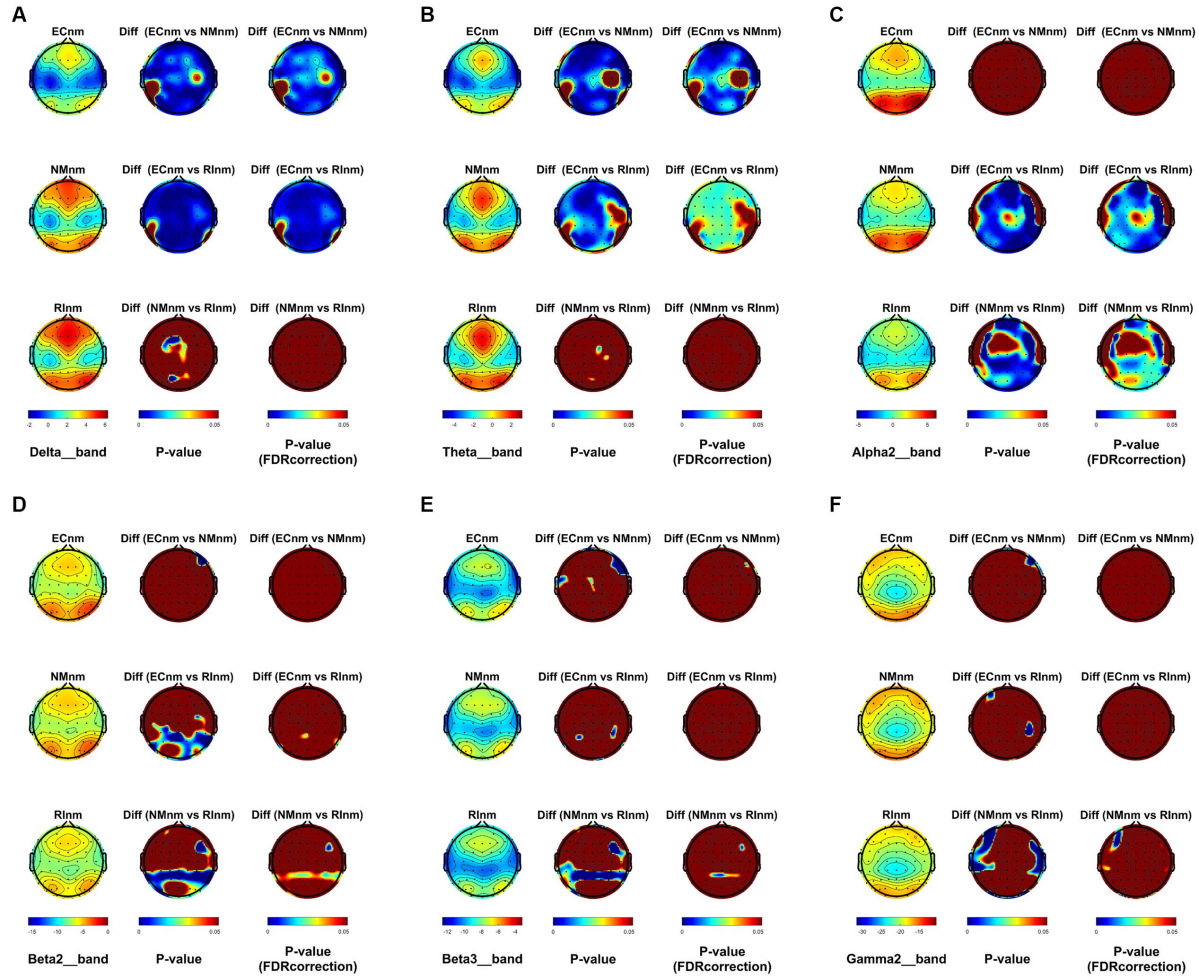
Scalp topographies of spectral analysis in TMNMT group. **(A–F)** Comparison of ECnm, NMnm, RInm of delta **(A)**, theta **(B)**, alpha2 **(C)**, beta2 **(D)**, beta3 **(E)**, and gamma2 **(F)** bands. The first column showed the PSD across the whole brain within each subgroup, the second and third column showed the uncorrected p values and corrected p values for multiple comparisons of a false discovery rate (FDR) across the whole brain within each two subgroups, respectively. ECnm, EEG recordings with eyes closed stimuli-pre by TMNMT music; NMnm, EEG recordings with eyes closed stimuli-ing by TMNMT music; RInm, EEG recordings with eyes closed stimuli-post by TMNMT music.

**Figure 7:**
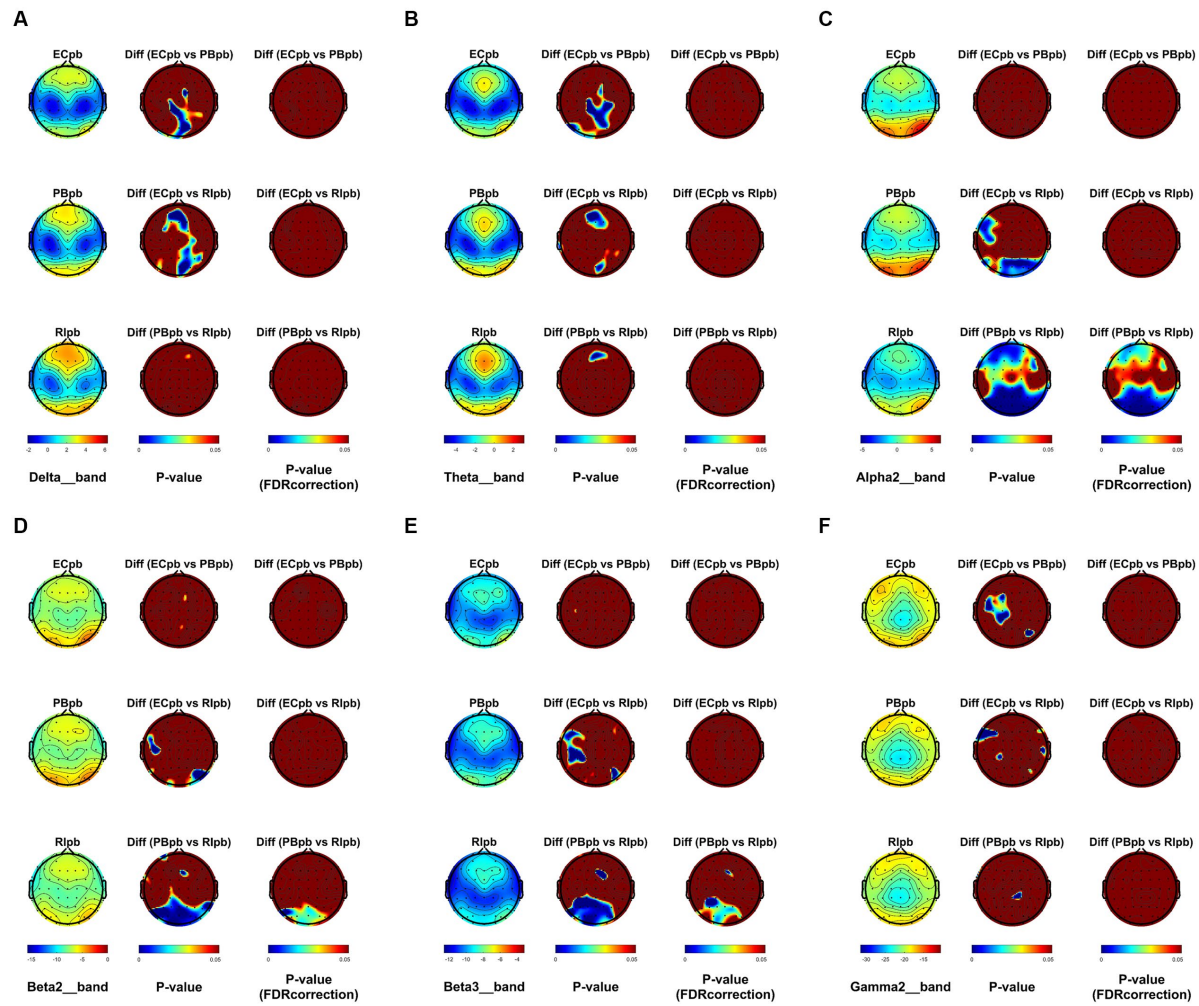
Scalp topographies of spectral analysis in Placebo group. **(A–F)** Comparison of ECpb, PBpb, RIpb of delta **(A)**, theta **(B)**, alpha2 **(C)**, beta2 **(D)**, beta3 **(E)**, and gamma2 **(F)** bands. The first column showed the PSD across the whole brain within each subgroup, the second and third column showed the uncorrected p values and corrected p values for multiple comparisons of a false discovery rate (FDR) across the whole brain within each two subgroups, respectively. ECpb, EEG recordings with eyes closed stimuli-pre by Placebo music; PBpb, EEG recordings with eyes closed stimuli-ing by Placebo music; RIpb, EEG recordings with eyes closed stimuli-post by Placebo music.

From the scalp topographies, through paired t-test analysis, the PSD of delta band and theta band of TMNMT group increased significantly, both ECnm vs. NMnm and ECnm vs. RInm had significant differences, and these effects survived correction for multiple comparisons of a false discovery rate (FDR) (Figures 6A,B); the PSD of alpha2 band, both ECnm vs. RInm and NMnm vs. RInm decreased significantly, and these effects remained after correction for multiple comparisons (Figure 6C); the PSD of beta2 band of ECnm vs. RInm decreased significantly, but this effect was nonexistent after correction for multiple comparisons of FDR and the PSD of beta2 band of NMnm vs. RInm decreased significantly, and this effect survived correction for multiple comparisons in part regions (Figure 6D); the PSD of beta3 band (NMnm vs. RInm) and gamma2 band (NMnm vs. RInm) decreased significantly, but these effects were nonexistent after correction for multiple comparisons of FDR (Figures 6E,F). In Placebo group, the PSD of delta band and theta band of ECpb vs. PBpb and ECpb vs. RIpb increased significantly in part regions, but these effects were nonexistent after correction for multiple comparisons of FDR (Figures 7A,B); the PSD of alpha2 band of ECpb vs. RIpb decreased significantly, but this effect was nonexistent after correction for multiple comparisons of FDR and the PSD of alpha2 band of PBpb vs. RIpb decreased significantly, and this effect was survival correction for multiple comparisons (Figure 7C); the PSD of beta2 band and beta3 band of PBpb vs. RIpb decreased significantly, and these effects survived correction for multiple comparisons in part regions (Figures 7D,E); the PSD of gamma2 band of ECpb vs. PBpb decreased significantly but this effect was nonexistent after correction for multiple comparisons of FDR (Figure 7F).

### 3.4 Microstate analysis

#### 3.4.1 Microstate topographies

**Figure 8:**
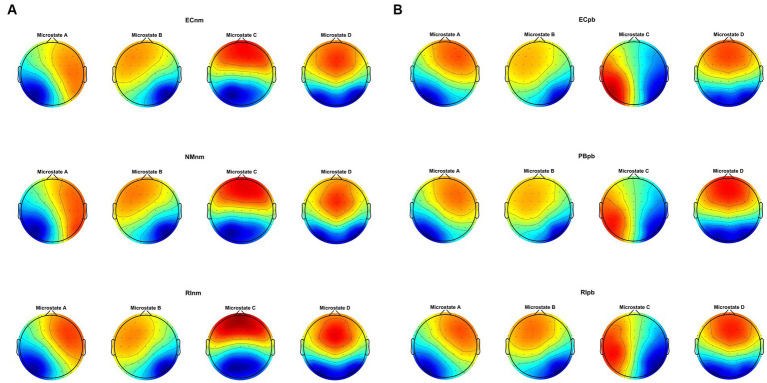
The spatial configuration of the four microstate topographies, separately for **(A)** TMNMT group and **(B)** Placebo group. Each row showed the three subgroups of TMNMT group and Placebo group. Each column showed the four topographic configurations (microstate A, B, C, D) for each subgroup. ECnm, EEG recordings with eyes closed stimuli-pre by TMNMT music; NMnm, EEG recordings with eyes closed stimuli-ing by TMNMT music; RInm, EEG recordings with eyes closed stimuli-post by TMNMT music; ECpb, EEG recordings with eyes closed stimuli-pre by Placebo music; PBpb, EEG recordings with eyes closed stimuli-ing by Placebo music; RIpb, EEG recordings with eyes closed stimuli-post by Placebo music.

Microstate analysis revealed that global clustering in TMNMT group (ECnm, NMnm, RInm subgroups) and Placebo group (ECpb, PBpb, RIpb subgroups) generated four microstates, that labeled Microstate A to D. In all groups, the four topographies of microstate classes A, B, C, and D were in accordance with those in previous reports (Lehmann et al., 1998; Custo et al., 2017; Michel and Koenig, 2018; Musaeus et al., 2019; Tait et al., 2020). Scalp topographies exhibited a left posterior-right anterior orientation (Microstate A), a right posterior -left anterior orientation (Microstate B), an anterior–posterior orientation (Microstate C), and a fronto-central maximum (Microstate D), respectively (Figure 8A). Moreover, the topography of microstate class C of ECpb, PBpb and RIpb subgroups was similar to that reported by Qin et al. (2022) and Bréchet et al. (2019), that exhibited in the left middle frontal gyrus, the dorsal part of anterior cingulate and cuneus extending to the posterior cingulate cortex, and thalamus (Custo et al., 2017) (Figure 8B).

#### 3.4.2 Microstate metrics

Repeated measures ANOVA was conducted to compare the duration, occurrence, coverage and transition possibility of the four microstates between TMNMT group and Placebo group. Microstate classes A, B, C and D were regarded as a within-subject factor and group as a between-subject factor.

**Figure 9:**
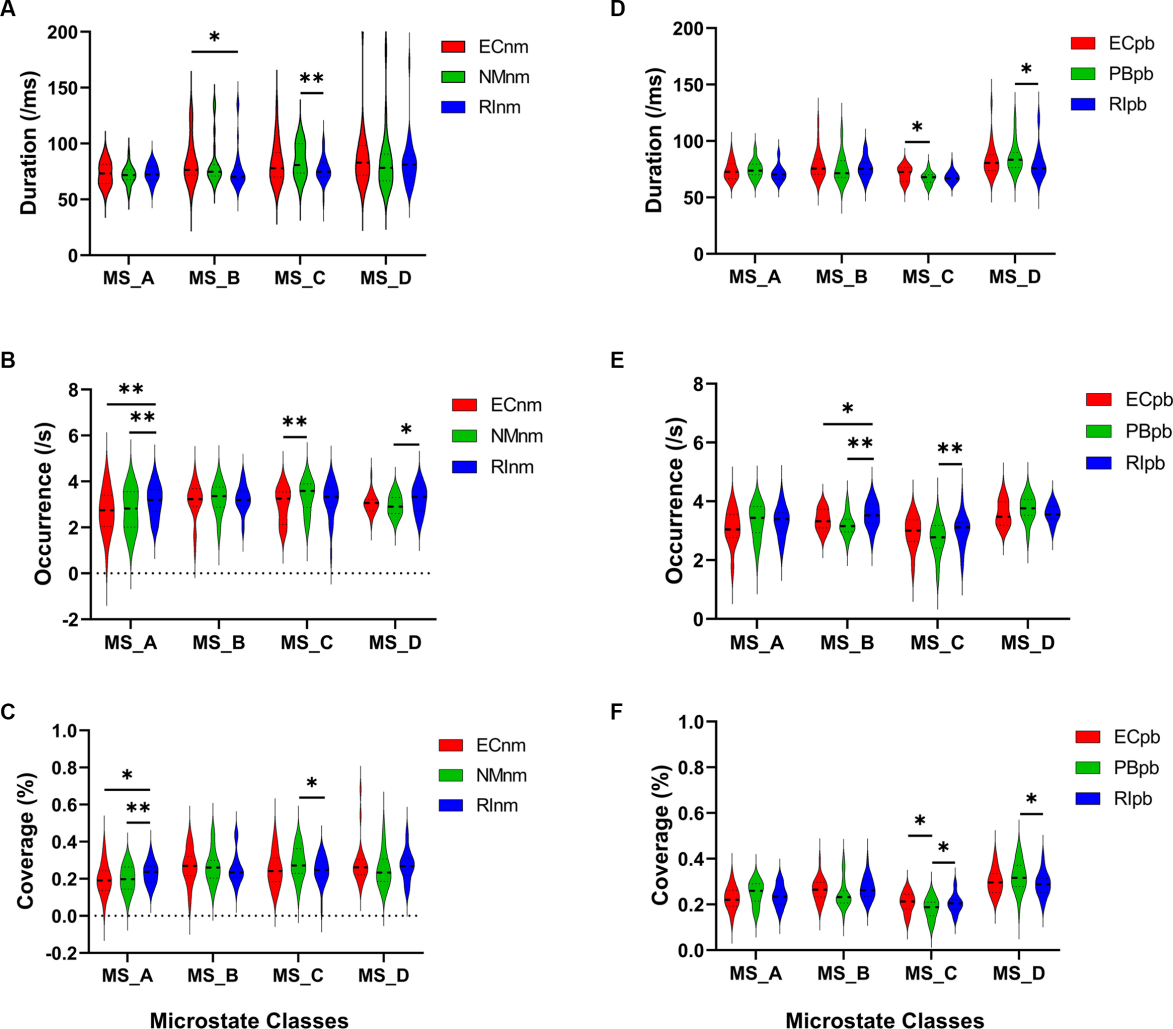
Microstate analysis of temporal metrics results: duration, occurrence, and coverage. Violin plots show each microstate class in the three subgroups of TMNMT and Placebo groups. **(A)** Microstate B of RInm had a shorter duration compared to ECnm, microstate C of RInm had a shorter duration compared to NMnm. **(B)** Microstate A of RInm had a higher occurrence compared to ECnm and NMnm, microstate C of NMnm had a higher occurrence compared to ECnm, and microstate D of RInm had a higher occurrence compared to NMnm. **(C)** microstate A of RInm had a higher coverage compared to ECnm and NMnm, microstate C of RInm had a lower coverage compared to NMnm. **(D)** Microstate C of PBpb had a shorter duration compared to ECpb, and microstate D of RIpb had a shorter duration compared to PBpb. **(E)** Microstate B of RIpb had a higher occurrence compared to ECpb and PBpb, microstate C of RIpb had a higher occurrence compared to PBpb. **(F)** Microstate C of PBpb had a lower coverage compared to ECpb and RIpb had a higher coverage compared to PBpb, microstate D of RIpb had a lower coverage compared to PBpb. ECnm, EEG recordings with eyes closed stimuli-pre by TMNMT music; NMnm, EEG recordings with eyes closed stimuli-ing by TMNMT music; RInm, EEG recordings with eyes closed stimuli-post by TMNMT music; ECpb, EEG recordings with eyes closed stimuli-pre by Placebo music; PBpb, EEG recordings with eyes closed stimuli-ing by Placebo music; RIpb, EEG recordings with eyes closed stimuli-post by Placebo music. *p < 0.05, **p < 0.01.

In TMNMT group, the results of microstate duration showed that the Microstate ∗ Group interaction effect (*F* = 2.192; *p* = 0.0461) and the main effect of group factor (*F* = 6.311; *p* = 0.00072) were significant, while the main effect of microstate factor was not significant (*F* = 2.298; *p* = 0.0834). Post-hoc comparisons showed microstate B of RInm subgroup had a shorter duration compared to ECnm subgroup (*p* = 0.0208), microstate C of RInm subgroup had a shorter duration compared to NMnm subgroup (*p* = 0.0041) (Table 4 and Figure 9A).

**Table 4:** The duration, occurrence, coverage of microstate (A–D) in TMNMT group (ECnm, NMnm, RInm).

Duration /ms Mean (Std)	ECnm (*n* = 22)	NMnm (*n* = 22)	RInm(*n* = 22)	*t*-value/*p*-value (ECnm – NMnm)	*t*-value/*p*-value (ECnm – RInm)	*t*-value/*p*-value (NMnm – RInm)
A	73.05 (10.00)	71.51 (7.74)	73.33 (7.42)	0.8349/0.6507	0.1728/0.9077	1.5840/0.4040
B	83.91 (19.11)	81.59 (19.51)	78.57 (20.30)	0.8808/0.4078	2.5000/0.0208	2.1640/0.0664
C	83.13 (17.96)	84.71 (14.20)	76.02 (10.86)	0.4640/0.4532	1.9170/0.0724	3.5330/0.0041
D	94.26 (40.08)	83.95 (27.01)	83.85 (22.99)	1.7700/0.0913	1.8430/0.0795	0.0530/0.9583

Occurrence/s Mean (Std)	ECnm (*n* = 22)	NMnm (*n* = 22)	RInm(*n* = 22)	*t*-value/*p*-value (ECnm – NMnm)	*t*-value/*p*-value (ECnm – RInm)	*t*-value/*p*-value (NMnm – RInm)
A	2.67 (0.91)	2.76 (0.87)	3.14 (0.67)	0.6728/0.1779	3.3720/0.0015	3.8840/0.0009
B	3.12 (0.79)	3.23 (0.66)	3.24 (0.56)	1.0660/0.2985	1.0570/0.3023	0.1161/0.9087
C	2.99 (0.69)	3.33 (0.67)	3.17 (0.79)	3.6060/0.0035	1.6220/0.1196	1.4830/0.1531
D	3.05 (0.42)	2.97 (0.44)	3.22 (0.57)	0.8151/0.2969	1.2340/0.2424	2.8630/0.0196

Coverage (%) Mean (Std)	ECnm (*n* = 22)	NMnm (*n* = 22)	RInm(*n* = 22)	*t*-value/*p*-value (ECnm – NMnm)	*t*-value/*p*-value (ECnm – RInm)	*t*-value/*p*-value (NMnm – RInm)
A	0.20 (0.08)	0.20 (0.07)	0.23 (0.06)	0.0179/0.3451	2.3820/0.0140	3.5970/0.0018
B	0.27 (0.09)	0.27 (0.09)	0.26 (0.08)	0.0159/0.9875	0.8688/0.3948	1.1030/0.2823
C	0.25 (0.09)	0.28 (0.08)	0.24 (0.07)	2.0830/0.0522	0.4336/0.4683	3.0570/0.0126
D	0.28 (0.12)	0.25 (0.09)	0.27 (0.08)	2.0380/0.1711	0.9422/0.3746	1.6640/0.1747

ECnm, EEG recordings with eyes closed stimuli-pre by TMNMT music; NMnm, EEG recordings with eyes closed stimuli-ing by TMNMT music; RInm, EEG recordings with eyes closed stimuli-post by TMNMT music.

The results of microstate occurrence showed that the Microstate ∗ Group interaction effect (*F* = 3.171; *p* = 0.0057) and the main effect of group factor (*F* = 8.912; *p* = 0.0003) were significant, while the main effect of microstate factor was not significant (*F* = 1.28; *p* = 0.2866). Post-hoc comparisons showed microstate A of RInm subgroup had a higher occurrence compared to ECnm subgroup (*p* = 0.0015) and NMnm subgroup (*p* = 0.0009), microstate C of NMnm subgroup had a higher occurrence compared to ECnm subgroup (*p* = 0.0035), microstate D of RInm subgroup had a higher occurrence compared to NMnm subgroup (*p* = 0.0196) (Table 4 and Figure 9B).

For the coverage of microstate, the Microstate ∗ Group interaction effect (*F* = 4.219; *p* = 0.0006) and the main effect of microstate factor (*F* = 2.744; *p* = 0.0481) were significant, while the main effect of group factor was not significant (*F*  =  0; *p* = 1). Post-hoc comparisons showed microstate A of RInm subgroup had a higher coverage compared to ECnm subgroup (*p* = 0.014) and NMnm subgroup (*p* = 0.0018), microstate C of RInm subgroup had a lower coverage compared to NMnm subgroup (*p* = 0.0126) (Table 4 and Figure 9C).

**Figure 10:**
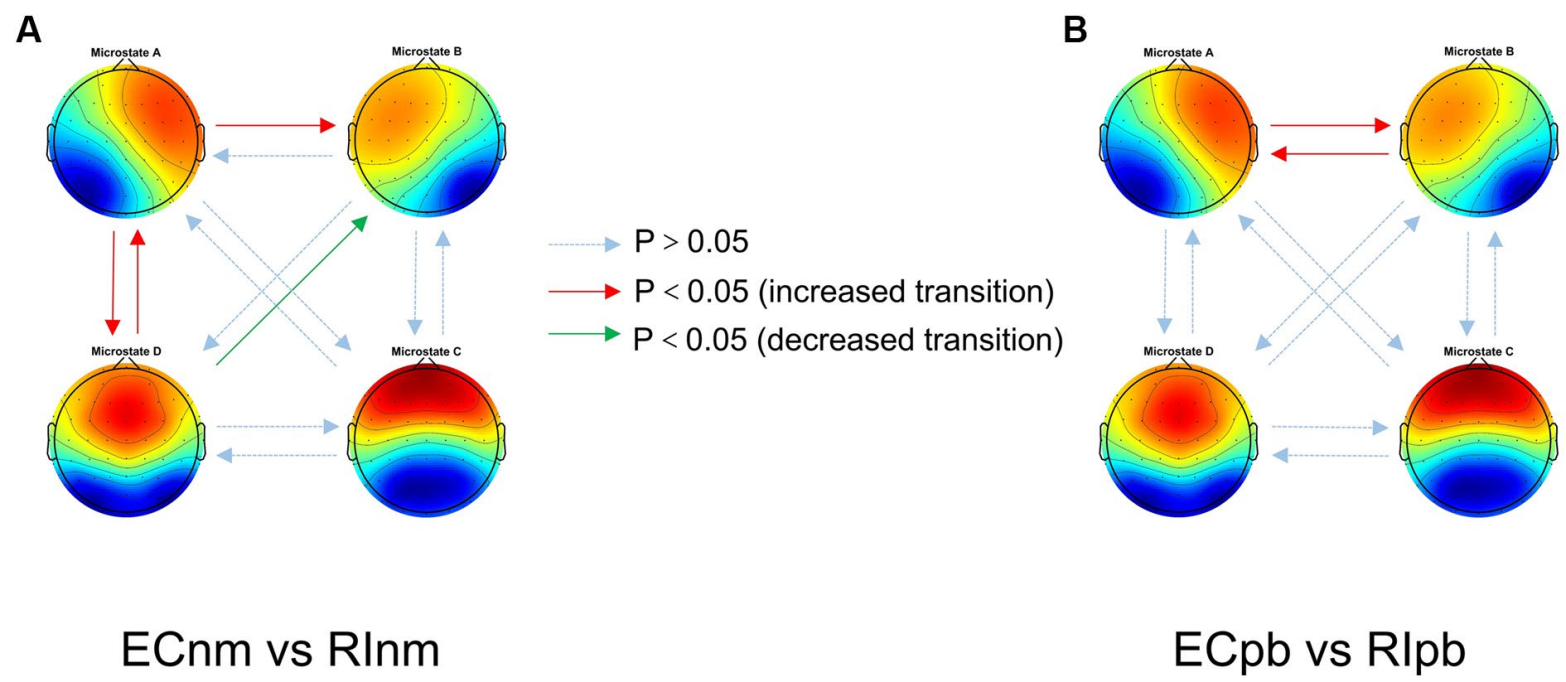
Schematic view of microstate syntax analysis results. A significant difference in transition probabilities for each pair of microstate class between ECnm subgroup and RInm subgroup **(A)**, ECpb subgroup and RIpb subgroup **(B)**. ECnm, EEG recordings with eyes closed stimuli-pre by TMNMT music; RInm, EEG recordings with eyes closed stimuli-post by TMNMT music; ECpb, EEG recordings with eyes closed stimuli-pre by Placebo music; RIpb, EEG recordings with eyes closed stimuli-post by Placebo music.

In terms of transition probabilities, we compared ECnm subgroup with RInm subgroup. The Microstate ∗ Group interaction effect (*F* = 4.31; *p* < 0.0001) and the main effect of microstate factor (*F* = 3.216; *p* = 0.0004) were significant, while the main effect of group factor was not significant (*F* = 0; *p* = 1). Post-hoc comparisons showed there were a lower possibility of transition from “D to B” in RInm subgroup compared to ECnm subgroup (*p* = 0.0344), a higher possibility of transition from “A to B” (*p* = 0.0452), “A to D” (*p* = 0.0054) and “D to A” (*p* = 0.0404) in RInm subgroup compared to ECnm subgroup (Table 6 and Figure 10A).

In Placebo group, the results of microstate duration showed that the Microstate ∗ Group interaction effect was not significant (*F* = 1.765; *p* = 0.1090), and the main effect of microstate factor (*F* = 10.40; *p* < 0.0001) and group factor (*F* = 4.003; *p* = 0.0245) were significant. Post-hoc comparisons showed microstate C of PBpb subgroup had a shorter duration compared to ECpb subgroup (*p* = 0.0093), microstate D of RIpb subgroup had a shorter duration compared to PBpb subgroup (*p* = 0.0169) (Table 5 and Figure 9D).

**Table 5:** The duration, occurrence, coverage of microstate (A–D) in Placebo group (ECpb, PBpb, RIpb).

Duration /ms Mean (Std)	ECpb(*n* = 22)	PBpb (*n* = 22)	RIpb(*n* = 22)	*t*-value/*p*-value (ECpb – PBpb)	*t*-value/*p*-value (ECpb – RIpb)	*t*-value/*p*-value (PBpb – RIpb)
A	73.81 (7.48)	75.07 (7.45)	71.57 (6.93)	1.1220/0.2883	2.0120/0.0902	2.5820/0.0547
B	78.70 (12.38)	76.11 (12.59)	76.36 (8.73)	1.1970/0.2445	0.9764/0.3400	0.1365/0.8927
C	71.04 (6.31)	67.06 (5.08)	68.23 (4.57)	3.1900/0.0093	2.2540/0.0852	1.2640/0.1540
D	83.63 (14.27)	86.01 (13.48)	80.65 (14.31)	0.8500/0.4049	0.9673/0.3444	2.9260/0.0169

Occurrence/s Mean (Std)	ECpb(*n* = 22)	PBpb(*n* = 22)	RIpb (*n* = 22)	*t*-value/*p*-value (ECpb – PBpb)	*t*-value/*p*-value (ECpb – RIpb)	*t*-value/*p*-value (PBpb – RIpb)
A	3.09 (0.58)	3.31 (0.56)	3.31 (0.50)	2.0950/0.0800	2.0720/0.0800	0.0756/0.9874
B	3.37 (0.34)	3.19 (0.37)	3.54 (0.42)	2.2730/0.0821	2.8280/0.0159	3.5890/0.0054
C	2.93 (0.53)	2.71 (0.55)	2.99 (0.53)	1.9490/0.0680	0.5058/0.4328	3.9840/0.0014
D	3.60 (0.42)	3.74 (0.43)	3.58 (0.30)	2.3700/0.0864	0.2115/0.8763	2.0230/0.0881

Coverage (%) Mean (Std)	ECpb(*n* = 22)	PBpb(*n* = 22)	RIpb(*n* = 22)	*t*-value/*p*-value (ECpb – PBpb)	*t*-value/*p*-value (ECpb – RIpb)	*t*-value/*p*-value (PBpb – RIpb)
A	0.23 (0.05)	0.25 (0.05)	0.24 (0.04)	2.2680/0.1070	1.1350/0.2691	1.3120/0.2036
B	0.26 (0.05)	0.25 (0.06)	0.27 (0.05)	1.7600/0.0929	0.5165/0.6415	1.9140/0.1464
C	0.21 (0.04)	0.18 (0.04)	0.20 (0.04)	2.6940/0.0071	0.3832/0.2469	3.1610/0.0049
D	0.30 (0.05)	0.32 (0.06)	0.29 (0.05)	2.0030/0.0612	0.9268/0.2552	2.9510/0.0160

ECpb, EEG recordings with eyes closed stimuli-pre by Placebo music; PBpb, EEG recordings with eyes closed stimuli-ing by Placebo music; RIpb, EEG recordings with eyes closed stimuli-post by Placebo music.

The results of microstate occurrence showed that the Microstate ∗ Group interaction effect (*F* = 4.853; *p* = 0.0001), the main effect of microstate factor (*F* = 13.03, *p* < 0.0001) and the main effect of group factor (*F* = 4.055; *p* = 0.0199) were significant. Post-hoc comparisons showed microstate B of RIpb subgroup had a higher occurrence compared to ECpb subgroup (*p* = 0.0159) and PBpb subgroup (*p* = 0.0054), microstate C of RIpb subgroup had a higher occurrence compared to PBpb subgroup (*p* = 0.0014) (Table 5 and Figure 9E).

For the coverage of microstate, the Microstate ∗ Group interaction effect (*F* = 4.811; *p* = 0.0001) and the main effect of microstate factor (*F* = 24.27; *p* < 0.0001) were significant, while the main effect of group factor was not significant (*F* = 0; *p* = 1). *Post-hoc* comparisons showed microstate C of PBpb subgroup had a lower coverage compared to ECpb subgroup (*p* = 0.0071) and RIpb subgroup had a higher coverage compared to PBpb subgroup (*p* = 0.0049), microstate D of RIpb subgroup had a lower coverage compared to PBpb subgroup (*p* = 0.0160) (Table 5 and Figure 9F).

In terms of transition probabilities, we compared ECpb subgroup with RIpb subgroup. The Microstate ∗ Group interaction effect (*F* = 4.812; *p* < 0.0001) and the main effect of microstate factor (*F* = 24.31; *p* < 0.0001) were significant, while the main effect of group factor was not significant (*F* = 0; *p* = 1). Post-hoc comparisons showed there were a higher possibility of transition from “A to B” (*p* = 0.0026) and “B to A” (*p* = 0.0061) in RIpb subgroup compared to ECpb subgroup (Table 6 and Figure 10B).

**Table 6:** Transition probabilities from one microstate class to all other classes in TMNMT and Placebo groups.

Transition probabilityMean (Std)	TMNMT			Placebo		
	ECnm (*n* = 22)	RInm(*n* = 22)	*t*-value/*p*-value (ECnm – RInm)	ECpb(*n* = 22)	RIpb(*n* = 22)	*t*-value/*p*-value (ECpb – RIpb)
A to B	0.30 (0.10)	0.34 (0.07)	2.1530/0.0452	0.27 (0.06)	0.30 (0.05)	3.4390/0.0026
A to C	0.29 (0.08)	0.32 (0.07)	1.5220/0.2251	0.33 (0.05)	0.34 (0.06)	0.2986/0.8066
A to D	0.26 (0.09)	0.31 (0.05)	2.8200/0.0054	0.34 (0.06)	0.34 (0.05)	0.4693/0.2253
B to A	0.36 (0.10)	0.35 (0.09)	0.5534/0.9227	0.29 (0.04)	0.32 (0.06)	3.0730/0.0061
B to C	0.32 (0.08)	0.33 (0.07)	0.6489/0.5496	0.35 (0.06)	0.35 (0.05)	0.1130/0.3189
B to D	0.38 (0.10)	0.35 (0.09)	1.9410/0.2074	0.41 (0.08)	0.40 (0.07)	0.5060/0.6491
C to A	0.35 (0.09)	0.32 (0.08)	1.2800/0.2045	0.31 (0.05)	0.31 (0.05)	0.2717/0.2760
C to B	0.32 (0.08)	0.31 (0.07)	0.1064/0.3207	0.31 (0.05)	0.29 (0.04)	1.3330/0.1377
C to D	0.36 (0.13)	0.35 (0.09)	0.7039/0.5137	0.25 (0.05)	0.26 (0.05)	0.3047/0.8018
D to A	0.30 (0.07)	0.33 (0.07)	2.2080/0.0404	0.41 (0.06)	0.38 (0.07)	1.7930/0.0612
D to B	0.38 (0.09)	0.34 (0.08)	2.2860/0.0344	0.43 (0.08)	0.41 (0.08)	1.4490/0.1701
D to C	0.39 (0.13)	0.36 (0.09)	1.6040/0.1948	0.31 (0.06)	0.31 (0.04)	0.4016/0.7266

ECnm, EEG recordings with eyes closed stimuli-pre by TMNMT music; RInm, EEG recordings with eyes closed stimuli-post by TMNMT music; ECpb, EEG recordings with eyes closed stimuli-pre by Placebo music; RIpb, EEG recordings with eyes closed stimuli-post by Placebo music.

All of the above value of ps effect had been corrected for multiple comparisons of FDR.

#### 3.4.3 Correlation between microstate metrics and TFI score

**Figure 11:**
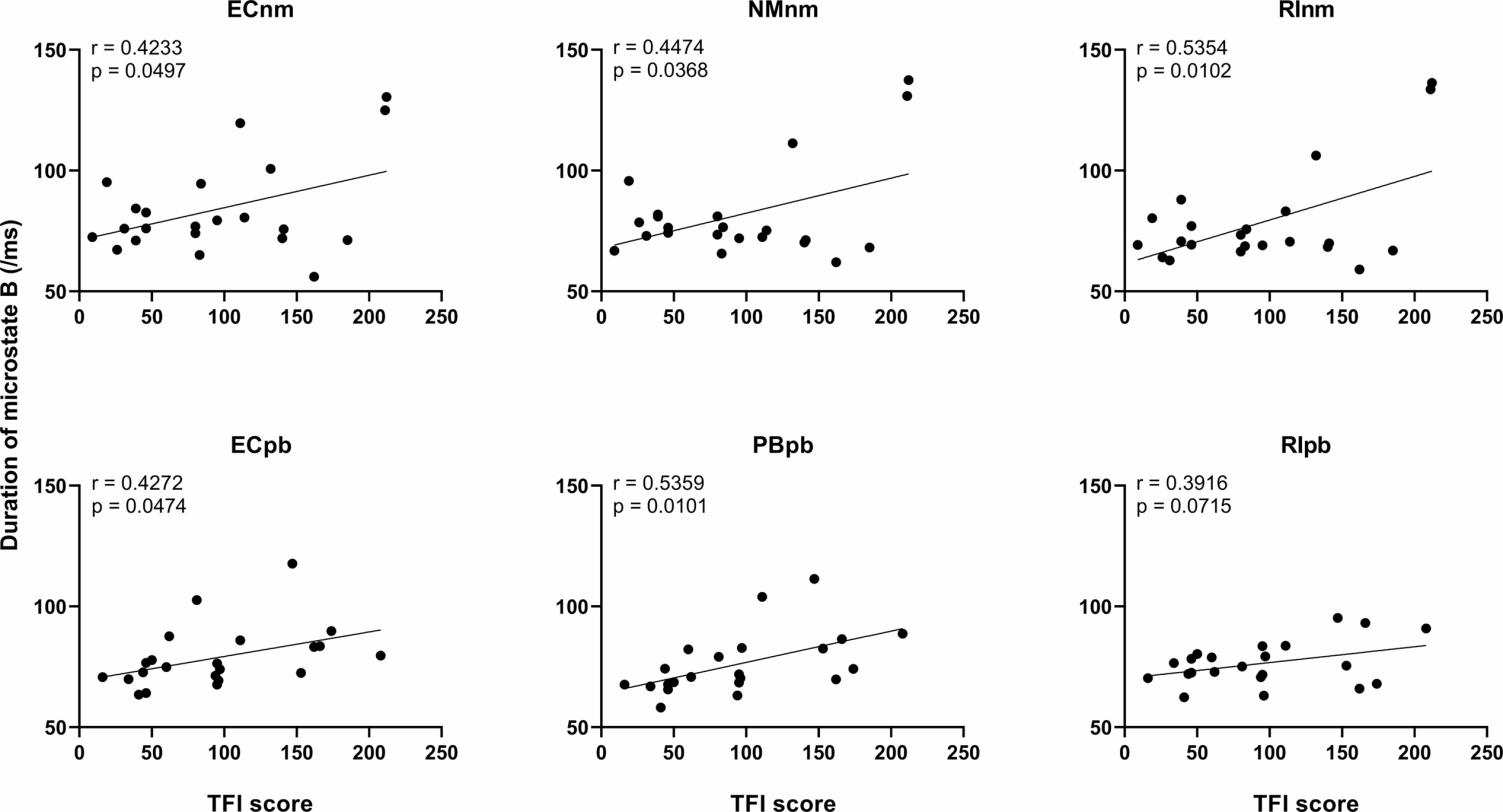
Correlation between the duration of microstate B and each subgroup. ECnm, EEG recordings with eyes closed stimuli-pre by TMNMT music; NMnm, EEG recordings with eyes closed stimuli-ing by TMNMT music; RInm, EEG recordings with eyes closed stimuli-post by TMNMT music; ECpb, EEG recordings with eyes closed stimuli-pre by Placebo music; PBpb, EEG recordings with eyes closed stimuli-ing by Placebo music; RIpb, EEG recordings with eyes closed stimuli-post by Placebo music.

We performed the Pearson correlation between microstate metrics and TFI score. The results revealed a significant positive correlation between the duration of microstate B and TFI score in ECnm subgroup (*r* = 0.4233, *p* = 0.0497), NMnm subgroup (*r* = 0.4474, *p* = 0.0368), RInm subgroup (*r* = 0.5354, *p* = 0.0102), ECpb subgroup (*r* = 0.4272, *p* = 0.0474), PBpb subgroup (*r* = 0.5359, *p* = 0.0101), and there was a positive correlation between the duration of microstate B and TFI score in RIpb subgroup, although it was not significant (*r* = 0.3916, *p* = 0.0715) (Figure 11). All of the above value of ps effect had been corrected for multiple comparisons of FDR.

## 4 Discussion

To our knowledge, the current study is the first to investigate the residual inhibition (RI) effect of the tailor-made notched music training (TMNMT), and analyze the power spectral density (PSD) and microstate classes using a resting-state EEG. We also included a placebo group as a control to ensure the credibility of the findings. To ensure the authenticity of the experiment and enhance the reliability of the study’s conclusions, we implemented rigorous criteria during the recruiting tinnitus subjects. Beyond meeting basic conditions, we specifically focused on the severity of tinnitus, preferring to enroll individuals experiencing severe tinnitus. For instance, in this study, the average THI and TFI scores for both groups exceeded 38 and 90, respectively (refer to Table 1). These thresholds, with a THI score of 38 and a TFI score of 50 generally considered as cutoff values indicative of tinnitus-related high distress, were chosen to obtain genuine and accurate data, thereby reinforcing the robustness of our study. These comprehensive approaches provided valuable insights into the effectiveness of TMNMT and enhances the novelty and significance of our research.

### 4.1 Whole-brain spectral analysis

**Figure 12:**
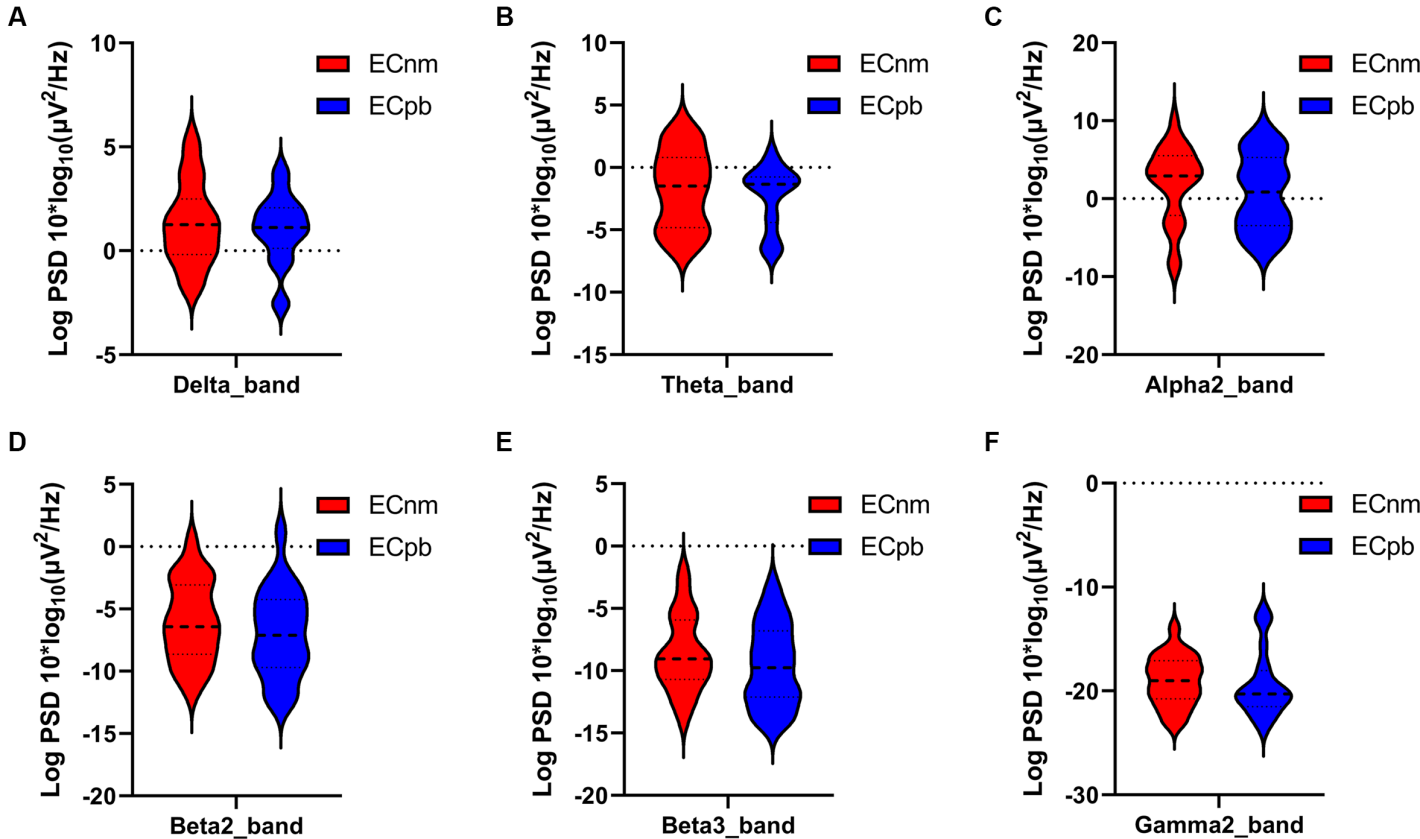
Waveforms of spectral analysis in ECnm vs. ECpb subgroups. **(A–F)** The PSD of delta **(A)**, theta **(B)**, alpha2 **(C)**, beta2 **(D)**, beta3 **(E)**, gamma2 **(F)** bands in ECnm vs. ECpb subgroups. ECnm, EEG recordings with eyes closed stimuli-pre by TMNMT music; ECpb, EEG recordings with eyes closed stimuli-pre by Placebo music.

**Figure 13:**
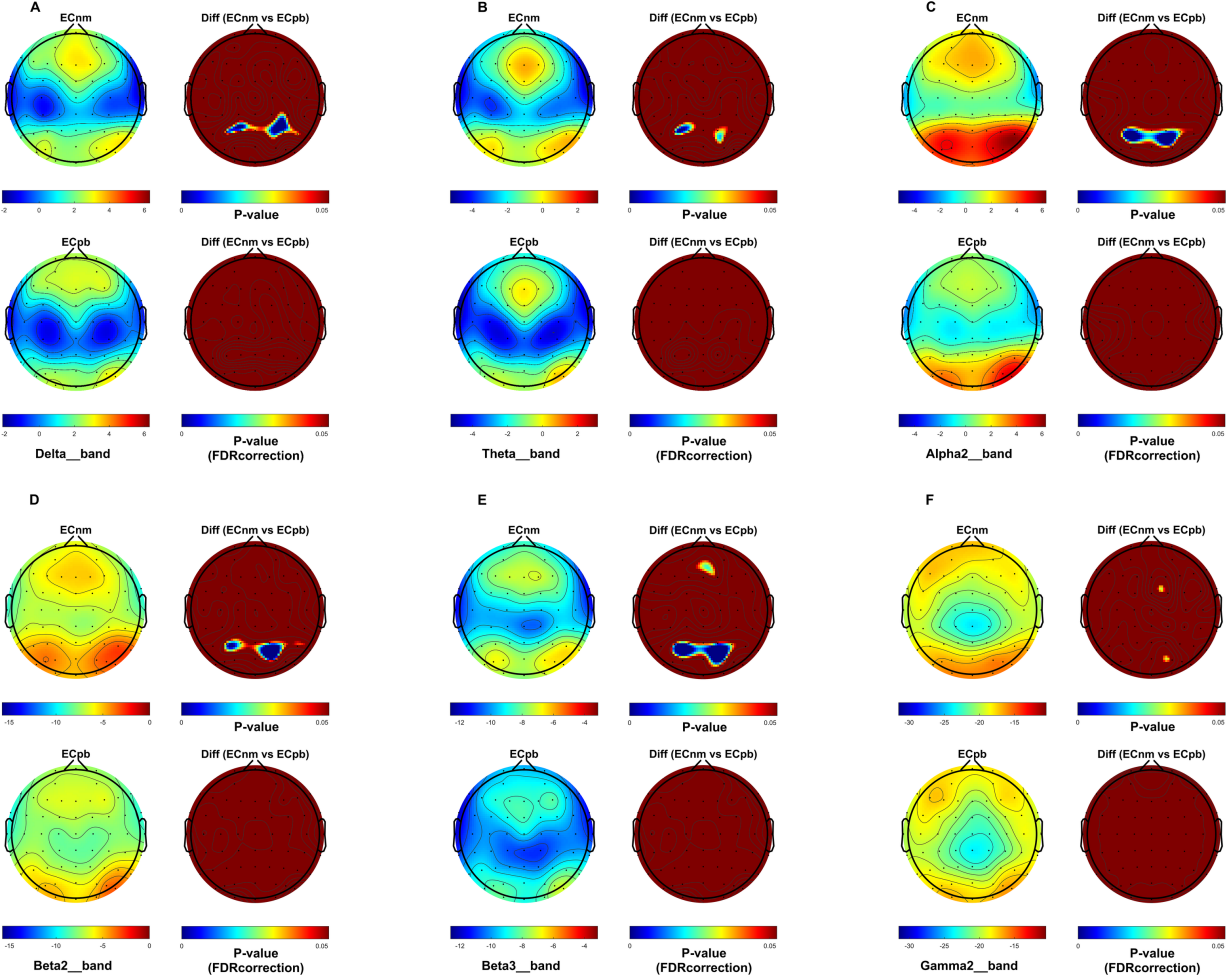
Scalp topographies of spectral analysis in ECnm vs. ECpb subgroups. **(A–F)** Comparison of ECnm, ECpb of delta **(A)**, theta **(B)**, alpha2 **(C)**, beta2 **(D)**, beta3 **(E)**, and gamma2 **(F)** bands. The first column showed the PSD across the whole brain within each subgroup, the first and second rows of the second column showed the uncorrected p values and corrected p values for multiple comparisons of a false discovery rate (FDR) across the whole brain within each two subgroups, respectively. ECnm, EEG recordings with eyes closed stimuli-pre by TMNMT music; ECpb, EEG recordings with eyes closed stimuli-pre by Placebo music.

To mitigate potential interference from other factors affecting the results, such as baseline differences or confounding variables, we conducted a comparison of the power spectral density (PSD) between the ECnm and ECpb subgroups. Figure 12 showed the power spectral density (PSD) of delta (*p* = 0.6335), theta (*p* = 0.4946), alpha2 (*p* = 0.4073), beta2 (*p* = 0.3652), beta3 (*p* = 0.3517) and gamma2 bands (*p* = 0.6560) between the ECnm and ECpb subgroups. The results revealed there were no significant differences in the above frequency bands (Table 7 and Figure 12). From the scalp topographies, the PSD of delta, theta, alpha2, beta2, beta3 and gamma2 bands were not found significant differences between the ECnm and ECpb subgroups (Figure 13).

**Table 7:** The power spectral density (PSD) of whole-brain certain frequency bands in ECnm vs. ECpb subgroups.

PSD (μV^2^/Hz)Mean (Std)	ECnm (*n* = 22)	ECpb (*n* = 22)	*t*-value	*p*-value
Delta	1.30 (1.94)	1.04 (1.63)	0.4803	0.6335
Theta	−1.75 (3.08)	−2.34 (2.43)	0.6891	0.4946
Alpha2	1.97 (4.74)	0.74 (4.75)	0.8370	0.4073
Beta2	−6.03 (3.19)	−6.97 (3.46)	0.9153	0.3652
Beta3	−8.66 (3.01)	−9.56 (3.22)	0.9417	0.3517
Gamma2	−19.00 (2.35)	−19.38 (3.13)	0.4486	0.6560

ECnm, EEG recordings with eyes closed stimuli-pre by TMNMT music; ECpb, EEG recordings with eyes closed stimuli-pre by Placebo music.

The results of this study showed that compared to stimuli-pre (ECnm), the PSD of delta band was significantly increased after TMNMT stimuli for 5 min (RInm), with a widespread distribution across the scalp, and no significant changes in Placebo group. Slow waves are a characteristic feature observed in cortical regions that lack connectivity with thalamocortical afferents (Steriade, 2006). slow waves at the cortical level can be enhanced by low-threshold spike bursts, generated in thalamic nuclei when hyperpolarized during deafferentation or overinhibition (Weisz et al., 2007). Delta activity is a characteristic oscillatory activity generated by deafferented/deprived neuronal networks (Llinás et al., 2005). RI may indicate the transient reestablishment of balance between excitatory and inhibitory neuronal assemblies through reafferentation (Eggermont and Tass, 2015). Increase of the oscillatory power in the delta, theta, as well as decrease in alpha power were associated with the presence of tinnitus and its intensity (Llinás et al., 1999, 2005; Weisz et al., 2005, 2007; Van der Loo et al., 2009; De Ridder et al., 2011; Adjamian et al., 2012; Tass et al., 2012; Adamchic et al., 2014; De Ridder et al., 2014, 2015b; Elgoyhen et al., 2015). An increase of oscillatory EEG power is typically interpreted as an increase in neuronal synchronization in terms of coincident firing within neuronal populations (Nunez, 1981 Bickford et al., 1979; Hämäläinen et al., 1993; Niedermeyer and da Silva, 2005). It was further found that an increase in delta activity was often accompanied by a decrease in other frequency spectrum, among the alpha band (Uchida et al., 1991; Benoit et al., 2000) as also detected in our study. Therefore, it is reasonable to speculate that the change of delta activity in tinnitus may be a multilateral balance state related to various spectral powers such as alpha wave, rather than a single event (Weisz et al., 2005). Thus, the enhancement of delta activity in tinnitus in our study compared to Placebo control group may represent a potential model that TMNMT was involved in the processing of tinnitus associated with auditory deafferentation, which would activate systems associated with tinnitus generation/cognition operating within the frequency range of delta activity (Weisz et al., 2005).

In the current study, we also found the PSD of theta band was significantly increased after TMNMT stimuli for 5 min (RInm), with a widespread distribution across the scalp, and no significant changes in Placebo group. Importantly, it has been proposed that low-frequencies modulate activity over large spatial regions in long temporal windows, whereas high frequencies modulate activity over small spatial regions and short temporal windows (Von Stein and Sarnthein, 2000). In other words, low frequencies (delta, theta, alpha) can be considered as carrier waves (Freeman and Rogers, 2002), and higher frequencies (beta, gamma) as information waves, and the higher frequencies are nested or carried by the lower frequencies. The theta wave acts as a long-range carrier wave (Freeman, 2005), hypothetically connecting to a theta oscillation-based memory network. The theta wave then acts as a compensatory mechanism to retrieve missing information from memory when it cannot be obtained from the external environment, as is the case for tinnitus patients with hearing loss (De Ridder et al., 2015b). Tinnitus, pain, movement, and mood-related information, via high-frequency oscillating activities such as beta and gamma, can be nested on this theta wave by means of cross-frequency coupling (De Ridder et al., 2014). Our findings supported the hypothesis that TMNMT-induced changes in theta band activity may play a compensatory role in tinnitus, facilitating the retrieval and integration of missing auditory information from memory networks. This cross-frequency coupling mechanism may provide a framework for understanding the complex interactions between different frequency bands in tinnitus pathophysiology.

The results of our study revealed a significant decrease in alpha2 band power in both TMNMT group and Placebo group, with a more pronounced effect observed in TMNMT group. In distressed tinnitus patients in contrast to controls low frequency activity (delta and theta) in the dorsal anterior cingulate cortex (dACC) is decreased, i.e., normal theta activity is decreased, and alpha1, alpha2 and beta3 activity is increased (Vanneste et al., 2010). In highly distressed vs. lowly distressed patients tend to show even higher levels of alpha1 and alpha2 activity, suggesting that the amount of alpha activity correlates with the level of distress (Vanneste et al., 2010). During our study, participants were instructed to focus their attention on the tinnitus sensation in order to provide intensity ratings. It is plausible that this attentional focus was enhanced after the sound stimuli, as participants had to be particularly attentive to detect any changes in intensity. This enhanced attentional state could have induced oscillatory brain activity that counteracted the effects induced by the stimulus alone. Indeed, reductions of alpha power have been frequently reported during tasks of focused attention (Jokisch and Jensen, 2007; Rihs et al., 2007). This fits well with the observation in our present study that alpha activity was reduced following the stimulus. Overall, our results suggest that TMNMT may modulate alpha2 band power, potentially influencing the attentional state and distress levels associated with tinnitus. These findings provide valuable insights into the complex relationship between oscillatory brain activity, attentional processes, and tinnitus perception.

In the current study, the results revealed a decrease of the PSD in beta2 and beta 3 bands in both TMNMT and Placebo groups, although the differences were not statistically significant in terms of topographical distribution. Studies suggest that reduced beta activity may serve functional inhibition (Jensen and Mazaheri, 2010; Müller et al., 2013). Several studies have reported that beta activity is associated with inhibitory states in the sensorimotor cortex, Hari and Salmelin (1997), Jensen et al. (2005), Miller et al. (2010), Schulz et al. (2014). Additionally, reduced power in the alpha up to the beta range has been linked to a stronger continuation of hearing music during noisy periods (Müller et al., 2013). Severe distress is associated with increased beta activity (Begić et al., 2001; Jokić-begić and Begić, 2003), which is consistent with a study that shows a connection between tinnitus distress and high beta (25 Hz) activity in frontal areas (Vanneste et al., 2010; Meyer et al., 2014).

The results revealed a decrease of the PSD in gamma2 band in TMNMT group, although the differences were not statistically significant in terms of topographical distribution. Tinnitus was suggested to be arise from abnormal, spontaneous, and constant gamma band activity generated as a consequence of the thalamic nuclei hyperpolarization (Llinás et al., 1999). The differential patterns of gamma-band oscillations in the auditory cortex associated with residual inhibition or excitation, suggesting a mutual inhibitory role of gamma oscillations on tinnitus symptoms (Sedley et al., 2012). These disparate findings may be attributed to the highly variable gamma band frequencies across studies (Weisz et al., 2007; Van Der Loo et al., 2009; Song et al., 2015) and confounders such as laterality and hearing loss (Ortmann et al., 2011). qEEG (Van Der Loo et al., 2009; Vanneste and De Ridder, 2012b; Meyer et al., 2014) and MEG studies (Weisz et al., 2005, 2007) showed a decrease in tinnitus loudness is associated with a decrease in gamma band activity in the auditory cortex (Vanneste and De Ridder, 2011; Adamchic et al., 2012, 2014) and a worsening in tinnitus loudness with an increase in auditory cortex gamma band activity (Vanneste et al., 2013). Gamma band activity increases and decreases were observed associated with an increase or decrease in tinnitus loudness following residual inhibition or excitation, respectively (Sedley et al., 2012), suggesting that gamma may represent a homeostatic change in loudness *per se*, with the tinnitus state as the reference, or it could potentially be attributed to other factors, such as associated hearing loss.

Through spectral analysis, it is evident that the delta, theta, and alpha bands hold potential as biomarkers in the TMNMT of tinnitus.

### 4.2 Microstate analysis

Microstate analysis explores multichannel EEG recordings across the scalp and defines a set of stable microstates (Khanna et al., 2015). Therefore, it not only compensates for the low spatial resolution of EEG signals to a certain extent, but also analyzes the abnormal dynamics of the whole brain networks. Resting state microstates reflects the temporarily independent functional network of the brain and the sum of spontaneous activity of neurons across various brain regions (Vanneste et al., 2019). Microstates are associated with spontaneous brain activity that are highly similar to fMRI resting state networks (Vanneste et al., 2019). The microstate analysis provides a unique perspective for studying the internal time dynamics of neuronal workspace models, but it has not been studied in thoroughly (Van de Ville et al., 2010). Current microstate analysis is mainly applied to schizophrenia, panic attacks, narcolepsy and depression (Kikuchi et al., 2011; Diaz Hernandez et al., 2016; Drissi et al., 2016; Zhang et al., 2018). Microstates had changed in chronic tinnitus patients and provided an indicator or perspective to explore the mechanisms of tinnitus. The maintenance of chronic subjective tinnitus may be related to changes in cerebral cortex activity (Cao et al., 2020). Studies suggests that the alterations of central neural networks occur in acute stage of hearing loss and tinnitus. And EEG microstate may be considered as a useful tool to study the whole brain network in ISSNHL patients (Cai et al., 2019), and EEG microstates may provide a possible valuable method to study large-scale brain networks, which may in turn be beneficial to investigation of the neurophysiological mechanisms behind tinnitus (Cai et al., 2018). Furthermore, Clinical studies have shown that alterations in brain activity may play an important role in the maintenance of tinnitus (Trakolis et al., 2018), a notion confirmed by findings from functional magnetic resonance imaging (fMRI) and EEG studies (Tian et al., 2011).

In the current study, the occurrence and coverage of microstate A significantly increased in TMNMT group. Microstate A is mainly associated with the changes of BOLD activation in the bilateral temporo-parietal cortex and meso-temporo-parietal cortex (Gschwind et al., 2016; Michel and Koenig, 2018). In addition, recent studies have estimated the sources of EEG microstates. The sources of microstate A are located on the left side of the occipital gyrus, insula, temporal lobe, and medial prefrontal cortex (mPFC) (Custo et al., 2017). Microstate A is closely related to auditory network and speech processing (Britz et al., 2010; Gschwind et al., 2016; Michel and Koenig, 2018). Therefore, the observed changes that a higher occurrence and coverage of microstate A in TMNMT (RInm vs. ECnm and RInm vs. NMnm) suggests that RI effect of TMNMT may enhance the auditory network and improve speech processing abilities in tinnitus patients.

Microstate B are significantly associated with BOLD changes in the striate and extrastriate cortex and the negative BOLD activation in the bilateral occipital cortex, which is closely related to the visual system (Britz et al., 2010; Yuan et al., 2012; Michel and Koenig, 2018). Moreover, microstate B is correlated with mental visualization of situations (Bréchet et al., 2019). A recent study showed that the presence of microstate B is associated with increased levels of anxiety (Damborská et al., 2019). In our study, a higher occurrence of microstate B in Placebo (RIpb vs. ECpb and RIpb vs. PBpb), along with a shorter duration of microstate B in TMNMT group (RInm vs. ECnm), suggest that RI effect of TMNMT may lead to a decrease in tinnitus intensity and alleviate the anxiety of tinnitus-related. These findings suggest that the impact of TMNMT extends beyond auditory processing, potentially involving the visual system and mental processes. The observed changes in microstate B duration provide valuable insights into the underlying mechanisms through which TMNMT exerts its effects on tinnitus-related anxiety and perceptual experiences.

Microstate C is associated with positive activation of BOLD in the bilateral inferior frontal cortex, dorsal anterior cingulate cortex, and right island area. Microstate C is thought to be generated at the bilateral medial temporal gyrus and the lateral parietal lobe (Wang et al., 2021). Microstate C is closely correlated with the default mode network (Britz et al., 2010; Gschwind et al., 2016; Michel and Koenig, 2018; Yu et al., 2021), which consists of the medial prefrontal, parietal and temporal cortex (Spreng et al., 2010). Default mode network is the neural basis of the ego (Andrews-Hanna et al., 2012); it has been found to be activated during internally-directed mental processes and to play an important role in self-referential thoughts and episodic memory retrieval (Xu et al., 2016; Michel and Koenig, 2018). Studies have shown that microstate C reflected the cognitive level of individuals with the disorders of consciousness (DOC). When the microstate C parameter of DOC patients is close to the normal level, it indicates an increased level of consciousness (Guo et al., 2022). In our study, a shorter duration with lower coverage of microstate C in PBpb subgroup (PBpb vs. ECpb), along with a higher occurrence and coverage of microstate C in RIpb subgroup (RIpb vs. PBpb), suggested that Placebo may impact levels of consciousness and cognition during stimuli, returning to baseline after stimuli. Similarly, a higher occurrence of microstate C in NMnm subgroup (NMnm vs. ECnm), along with a shorter duration and lower coverage of microstate C in RInm subgroup (RInm vs. NMnm), suggested that TMNMT may improve the level of consciousness and cognition during stimuli, returning to baseline after stimuli. The above statement implies that TMNMT has the potential to enhance cognitive processes and self-awareness of tinnitus patients; however, further research is required to validate this assertion.

Microstate D is associated with negative BOLD activation in the right ventral and dorsal regions of the frontal cortex and parietal cortex, which is closely related to attention network (Koenig et al., 1999; Gschwind et al., 2016; Michel and Koenig, 2018). Microstate D is widely connected to default mode and dorsal attention network (Spreng et al., 2013). Attention deficit has long been considered one of the symptoms of tinnitus. The psychological complications, such as annoyance, attention deficits, depression, anxiety, irritability, sleep disturbances, and intense worrying can be a result of constant awareness of tinnitus (Vanneste et al., 2010). In our study, a shorter duration and lower coverage of microstate D in Placebo group (RIpb vs. PBpb), along with a higher occurrence of microstate D in TMNMT group (RInm vs. NMnm) suggested that RI effect of TMNMT may enhance frontal parietal cortex and dorsal attention networks in tinnitus patients. By modulating the activity of the attention network, TMNMT has the potential to improve attentional processes and alleviate attention deficits in individuals with tinnitus. The increased occurrence of microstate D provides further evidence of the positive effects of TMNMT on attention networks, which may contribute to the overall improvement in attentional functioning and symptom management observed in our study.

A speculative point of view that we observed the microstate C in all three Placebo subgroups, which is similar to previous studies and referred to as microstate class E (Bréchet et al., 2019; Qin et al., 2022). Microstate class E is thought to be associated with the default mode network in the dorsal anterior cingulate cortex, which extends to the superior frontal gyrus, middle frontal gyrus, and insula (Custo et al., 2017). The activities of these networks are associated with the midcingulo-insular network regions (Uddin et al., 2019). The midcingulo-insular network is also thought to regulate the default mode network and participate in dynamic switching between large-scale related networks to facilitate attention and response to the external environment (Zanesco et al., 2021). Therefore, our findings suggest that the reduction in microstate C observed in Placebo subgroups may be attributed to impaired default mode network in individuals experiencing RI effect of the placebo treatment. The disrupted default mode network in Placebo group may contribute to alterations in attentional processes and the ability to shift between different brain networks. These findings provide insights into the potential mechanisms underlying the placebo effect and the involvement of default mode network dysfunction in tinnitus.

The study also identified several variations of transition probabilities among microstate classes between TMNMT and Placebo groups. Transition probabilities may indicate a switch between intrinsic brain function networks, as microstate classes are related to their corresponding intrinsic brain function networks (Britz et al., 2010; Milz et al., 2016; Custo et al., 2017; Michel and Koenig, 2018). We found that the transition of microstate class mainly involves microstate A in both TMNMT and Placebo groups, that indicate a heightened activity within the auditory networks. Increased transitions from microstates A to microstates B and microstates B to microstates A may implicate RI effect of Placebo enhances the mutual functional connectivity between the auditory network and the visual network. Increased transitions from microstates A to microstates B may implicate that RI effect of TMNMT enhanced functional connectivity from the auditory networks to visual networks. These findings suggest that RI of TMNMT and Placebo facilitated communication between these networks, potentially influencing the processing of auditory and visual information. An increase in the inter-transitions between microstates A and D and a reduction transition from microstates D to microstates B implicate that RI effect of TMNMT increased the functional connectivity of auditory and attention networks, while decreased the functional connectivity from the attention networks to visual networks. These changes in connectivity patterns suggest that RI of TMNMT may have a positive impact on the interaction between auditory and attention networks, potentially influencing cognitive processes related to attention and sensory information processing.

In addition, we observed the duration of microstate B were positive correlations of tinnitus participants’ TFI scores among six subgroups of TMNMT and Placebo groups. These results suggest that microstate B is associated with the intensity of tinnitus symptoms, which is consistent with the similar studies of Atluri et al. (2018) and Yan et al. (2021). Atluri et al. (2018) reported that a reduced occurrence of microstate B in patients with major depressive disorder following the treatment was associated with significant obvious clinical outcomes. Yan et al. (2021) found that the duration and occurrence of microstate B were significantly reduced in patients with major depression after treatment. Liang et al. (2021) also reported an increased occurrence of microstate B in the depression group compared to the healthy control group.

## 5 Limitations and future work

Inevitably, limitations of this study need to be mentioned. First, due to the widespread, intricate and heterogeneity of tinnitus, it may not be possible to process it in a homogeneous manner even with rigorous inclusion and exclusion criteria and carrying out the high experimental standards. The variability among tinnitus patients may have influenced the results and should be considered in future research. Second, understanding the cortical correlations of EEG microstates relies on source modeling techniques that provide limited spatial resolution at best. These methods rely on multiple biophysical assumptions and simplifications that are still undergoing refinement (Nunez and Srinivasan, 2006). We encourage future studies to amplify the transitions between resting EEG microstates in order to better understand the functions underlying the communication between neural resting networks on the millisecond scale. Third, As described earlier, to obtain the intensity and duration of residual inhibition, participants were always required to focus their attention on the tinnitus sensation. It is possible that attentional factors contributed to the observed oscillatory brain activity, which may have counteracted the effects induced solely by the stimuli. In future studies, a RI condition should be examined in order to minimize attention effects and thus, unspecific changes in alpha activity. It is recommended to test and validate the intensity and duration of RI prior to the experiment, and provide an estimation of tinnitus loudness during the course of the study. In summary, due to the heterogeneity of tinnitus and relatively small sample size, the results of our study require careful read and cautious interpretation. Hence, further studies with larger sample sizes are required to enhance sample homogeneity and reduce the heterogeneity present in tinnitus samples and a more comprehensive analysis is necessary to investigate the underlying mechanisms.

## 6 Conclusion

In this study, behavioral test, spectral analysis and EEG microstates were used to demonstrate that TMNMT, a novel therapy for tinnitus, had a stronger ability of residual inhibition, which provided valuable experimental evidence and practical implications for the potential applications of TMNMT in tinnitus treatment. Additionally, EEG microstate temporal dynamics may serve as novel functional state and trait markers of synchronous brain activity that contribute to a deep understanding of the neural mechanism underlying TMNMT treatment for tinnitus.

## Data availability statement

The raw data supporting the conclusions of this article will be made available by the authors, without undue reservation.

## Ethics statement

The studies involving humans were approved by the Medical Ethics Committee of Tsinghua University. The studies were conducted in accordance with the local legislation and institutional requirements. The participants provided their written informed consent to participate in this study.

## Author contributions

MZ: Writing – review & editing, Conceptualization, Data curation, Formal analysis, Investigation, Methodology, Writing – original draft. QG: Conceptualization, Writing – review & editing, Funding acquisition, Project administration, Resources, Software, Supervision, Validation, Visualization.

## Footnotes

^1^http://compumedicsneuroscan.com/

^2^http://www.thomaskoenig.ch/index.php/software/microstates-in-eeglab
