# Corrigendum: EEG spectral and microstate analysis originating residual inhibition of tinnitus induced by tailor-made notched music training

**DOI:** 10.3389/fnins.2023.1355150

**Published:** 2024-01-08

**Authors:** Min Zhu, Qin Gong

**Affiliations:** ^1^Department of Biomedical Engineering, School of Medicine, Tsinghua University, Beijing, China; ^2^School of Medicine, Shanghai University, Shanghai, China

**Keywords:** tinnitus, tailor-made notched music training, residual inhibition, EEG, spectral analysis, microstate

In the original article, there are two references cited incorrectly.

One is in the section 2.5 EEG data collection and pre-processing:

“To minimize potential interference from alcohol, coffee, cola, and tea on EEG recordings (Barry et al., 2011; Foxe et al., 2012; Vanneste and De Ridder, 2012b).” It should be To minimize potential interference from alcohol, coffee, cola, and tea on EEG recordings (Barry et al., 2011; Foxe et al., 2012; Vanneste and De Ridder, 2012a).

The reference is:

Vanneste, S., and De Ridder, D. (2012a). The use of alcohol as a moderator for tinnitusrelated distress. *Brain Topogr*. 25, 97–105. doi: 10.1007/s10548-011-0191-0

The other is in the section 4.1 Whole-brain spectral analysis:

“qEEG (Van Der Loo et al., 2009; Vanneste and De Ridder, 2012a; Meyer et al., 2014) and MEG studies··” It should be qEEG (Van Der Loo et al., 2009; Vanneste and De Ridder, 2012b; Meyer et al., 2014) and MEG studies··

The reference is:

Vanneste, S., and De Ridder, D. (2012b). The auditory and non-auditory brain areas involved in tinnitus. An emergent property of multiple parallel overlapping subnetworks. *Front. Syst. Neurosci*. 6:31. doi: 10.3389/fnsys.2012.00031

In the published article, for the keeping consistency of the whole article, the font of some words should be consistent, either in orthography or italics. Specifically as follows:

(1) Page 05, Section 2.7 Microstate analysis

The original sentence:


$$\begin{array}{l} GFP\left(t\right)\;=\sqrt{\frac{{\left[\sum_{i}^{N}\left(Vi\;\left(t\;\right)-Vmean\left(t\;\right)\right)\right]}^{2}}{N}} \end{array}$$

Where *N* represents the number of electrodes in the EEG data (*i* = 1: 60), *V* represents the electrical potential measured over the scalp. *Vi*(*t*) represents the instantaneous electrical potential at electrode *i* and time *t*, and *V*mean(*t*) represents the average electrical potential at all electrodes at time *t*.

The corrected sentence appears below:


$$\begin{array}{l} GFP\left(t\right)\;=\sqrt{\frac{{\left[\sum_{i}^{N}\left(Vi\left(t\right)-Vmean\left(t\right)\right)\right]}^{2}}{N}} \end{array}$$

Where N represents the number of electrodes in the EEG data (i = 1: 60), V represents the electrical potential measured over the scalp. Vi(t) represents the instantaneous electrical potential at electrode i and time t, and Vmean(t) represents the average electrical potential at all electrodes at time t.

(Authors considered that formula and text should keep consistency in font, it's either all in orthography or all in italics)

(2) Page 06, Section 3.3 Whole-brain spectral analysis

1) The original sentence:

Post-hoc comparisons showed the PSD of delta band (ECnm vs. NMnm, *p* = 0.0005; ECnm vs. RInm, p = 0.0005; Figure 4B)…

The corrected sentence appears below:

Post-hoc comparisons showed the PSD of delta band (ECnm vs. NMnm, *p* = 0.0005; ECnm vs. RInm, *p* = 0.0005; Figure 4B)…

2) The original sentence:

Figure 5A showed the PSD of whole-brain full bands in Placebo group, the results of PSD showed that the main effect of frequency band factor (*F* = 315.2; p < 0.0001) was significant,…

The corrected sentence appears below:

Figure 5A showed the PSD of whole-brain full bands in Placebo group, the results of PSD showed that the main effect of frequency band factor (*F* = 315.2; *p* < 0.0001) was significant,

3) The original sentence:

…beta3 band (PBpb vs. RIpb, p = 0.0027; Figure 5F) were significantly decreased in Placebo group (Table 3 and Figure 5).

The corrected sentence appears below:

…beta3 band (PBpb vs. RIpb, *p* = 0.0027; Figure 5F) were significantly decreased in Placebo group (Table 3 and Figure 5).

(3) Page 08, section 3.4.2 Microstate metrics

1) The original sentence:

The Microstate * Group interaction effect (*F* = 4.31; *p* < 0.0001) and the main effect of microstate factor (*F* = 3.216; *p* = 0.0004) were significant, while the main effect of group factor was not significant (F = 0; p = 1).

The corrected sentence appears below:

The Microstate * Group interaction effect (*F* = 4.31; *p* < 0.0001) and the main effect of microstate factor (*F* = 3.216; *p* = 0.0004) were significant, while the main effect of group factor was not significant (*F* = 0; *p* = 1).

2) The original sentence:

In Placebo group, the results of microstate duration showed that the Microstate * Group interaction effect was not significant (*F* = 1.765; *p* = 0.1090), and the main effect of microstate factor (*F* = 10.40; p < 0.0001) and group factor (*F* = 4.003; *p* = 0.0245) were significant.

The corrected sentence appears below:

In Placebo group, the results of microstate duration showed that the Microstate * Group interaction effect was not significant (*F* = 1.765; *p* = 0.1090), and the main effect of microstate factor (*F* = 10.40; *p* < 0.0001) and group factor (*F* = 4.003; *p* = 0.0245) were significant.

3) Page 09, The original sentence:

The results of microstate occurrence showed that the Microstate * Group interaction effect (*F* = 4.853; *p* = 0.0001), the main effect of microstate factor (*F* = 13.03, p < 0.0001) and the main effect of group factor (*F* = 4.055; *p* = 0.0199) were significant.

The corrected sentence appears below:

The results of microstate occurrence showed that the Microstate * Group interaction effect (*F* = 4.853; *p* = 0.0001), the main effect of microstate factor (*F* = 13.03, *p* < 0.0001) and the main effect of group factor (*F* = 4.055; *p* = 0.0199) were significant.

4) Page 09, The original sentence:

For the coverage of microstate, the Microstate * Group interaction effect (*F* = 4.811; p = 0.0001) and the main effect of microstate factor (*F* = 24.27; *p* < 0.0001) were significant, while the main effect of group factor was not significant (F = 0; p = 1).

The corrected sentence appears below:

For the coverage of microstate, the Microstate * Group interaction effect (*F* = 4.811; *p* = 0.0001) and the main effect of microstate factor (*F* = 24.27; *p* < 0.0001) were significant, while the main effect of group factor was not significant (*F* = 0; *p* = 1).

5) Page 09, The original sentence:

In terms of transition probabilities, we compared ECpb subgroup with RIpb subgroup. The Microstate * Group interaction effect (*F* = 4.812; p < 0.0001) and the main effect of microstate factor (*F* = 24.31; p < 0.0001) were significant, while the main effect of group factor was not significant (*F* = 0; *p* = 1).

The corrected sentence appears below:

In terms of transition probabilities, we compared ECpb subgroup with RIpb subgroup. The Microstate * Group interaction effect (*F* = 4.812; *p* < 0.0001) and the main effect of microstate factor (*F* = 24.31; *p* < 0.0001) were significant, while the main effect of group factor was not significant (*F* = 0; *p* = 1).

Page 10, Figure 6

The original sentence:

Scalp topographies of spectral analysis in TMNMT group. **(A–F)** Comparison of ECnm, NMnm, RInm of delta **(A)**, theta **(B)**, alpha2 **(C)**, beta2 **(D)**, beta3 **(E)**, and gamma2 **(F)** bands. The first column showed the PSD across the whole brain within each subgroup, the second and third column showed the uncorrected *p* values and corrected p values for multiple comparisons of a false discovery rate (FDR) across the whole brain within each two subgroups, respectively. ECnm, EEG recordings with eyes closed stimuli-pre by TMNMT music; NMnm, EEG recordings with eyes closed stimuli-ing by TMNMT music; RInm, EEG recordings with eyes closed stimuli-post by TMNMT music.

The corrected sentence appears below: (red mark “*p*” should be ortho “p”)

Scalp topographies of spectral analysis in TMNMT group. **(A–F)** Comparison of ECnm, NMnm, RInm of delta **(A)**, theta **(B)**, alpha2 **(C)**, beta2 **(D)**, beta3 **(E)**, and gamma2 **(F)** bands. The first column showed the PSD across the whole brain within each subgroup, the second and third column showed the uncorrected p values and corrected p values for multiple comparisons of a false discovery rate (FDR) across the whole brain within each two subgroups, respectively. ECnm, EEG recordings with eyes closed stimuli-pre by TMNMT music; NMnm, EEG recordings with eyes closed stimuli-ing by TMNMT music; RInm, EEG recordings with eyes closed stimuli-post by TMNMT music.

The authors apologize for the errors and state that these do not change the scientific conclusions of the article in any way. The original article has been updated.

