# Corrigendum: EEG spectral and microstate analysis originating residual inhibition of tinnitus induced by tailor-made notched music training

**DOI:** 10.3389/fnins.2024.1369080

**Published:** 2024-04-12

**Authors:** Min Zhu, Qin Gong

**Affiliations:** ^1^Department of Biomedical Engineering, School of Medicine, Tsinghua University, Beijing, China; ^2^School of Medicine, Shanghai University, Shanghai, China

**Keywords:** tinnitus, tailor-made notched music training, residual inhibition, EEG, spectral analysis, microstate

In the published article, there is an error in Figure 2B as published. The local maxima within the red dotted line was seven red dots, but should be eight red dots since a local maxima represents a topographic map. The corrected Figure 2 appears below.

**Figure 2:**
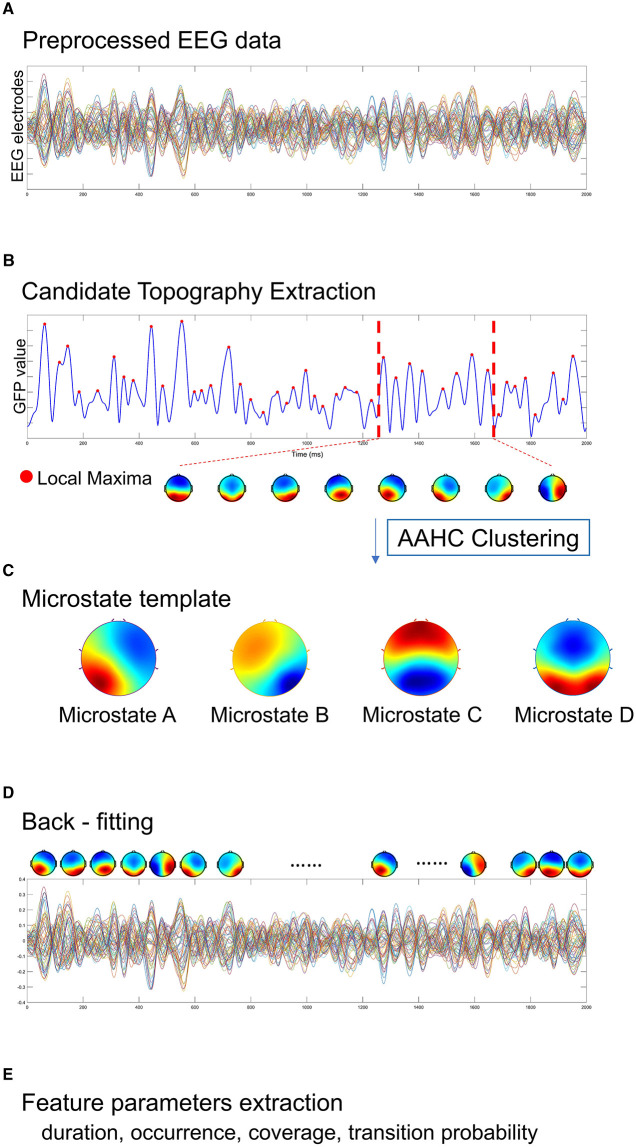
A standard procedure of EEG microstate analysis. Based on **(A)** the preprocessed EEG data, **(B)** candidate topographies with high signal-to-noise ratios were extracted from the local maxima of the GFP curve, **(C)** four templates were obtained after AAHC Clustering. **(D)** The final detected EEG microstate templates were then fitted back into the preprocessed EEG data by assigning each time point to a predominant microstate. After EEG microstates back-fitting, the original EEG time series were re-represented into EEG microstate sequences covering whole-brain spontaneous spatial–temporal activities. **(E)** A several of microstate feature metrics were calculated for quantitative measurement, including duration, occurrence, coverage, transition probability.

In addition, there was an error in the Acknowledgments section. “Xi Li” should be “Xin Li”. The corrected statement appears below:

We would like to thank Haijin Yi and Xin Li from Beijing Tsinghua Changgung Hospital for the participants' recommendation and thank all the participants who participated in the study.

The authors apologize for these errors and state that this does not change the scientific conclusions of the article in any way. The original article has been updated.

